# The diabetes drug liraglutide reverses cognitive impairment in mice and attenuates insulin receptor and synaptic pathology in a non‐human primate model of Alzheimer's disease

**DOI:** 10.1002/path.5056

**Published:** 2018-04-02

**Authors:** Andre F Batista, Leticia Forny‐Germano, Julia R Clarke, Natalia M Lyra e Silva, Jordano Brito‐Moreira, Susan E Boehnke, Andrew Winterborn, Brian C Coe, Ann Lablans, Juliana F Vital, Suelen A Marques, Ana MB Martinez, Matthias Gralle, Christian Holscher, William L Klein, Jean‐Christophe Houzel, Sergio T Ferreira, Douglas P Munoz, Fernanda G De Felice

**Affiliations:** ^1^ Institute of Medical Biochemistry Leopoldo de Meis Federal University of Rio de Janeiro Rio de Janeiro Brazil; ^2^ School of Pharmacy Federal University of Rio de Janeiro Rio de Janeiro Brazil; ^3^ Centre for Neuroscience Studies, Department of Biomedical and Molecular Sciences Queen's University Kingston Ontario Canada; ^4^ Animal Care Service Queen's University Kingston Ontario Canada; ^5^ Departament of Neurobiology Fluminense Federal University Niteroi Brazil; ^6^ Department of Pathology, Faculty of Medicine Hospital Universitário Clementino Fraga Filho, UFRJ Rio de Janeiro Brazil; ^7^ Division of Biomed and Life Sciences Faculty of Health and Medicine Lancaster University Lancaster UK; ^8^ Department of Neurobiology Northwestern University Evanston Illinois USA; ^9^ Institute of Biomedical Sciences Federal University of Rio de Janeiro Rio de Janeiro Brazil; ^10^ Institute of Biophysics Carlos Chagas Filho Federal University of Rio de Janeiro Rio de Janeiro Brazil

**Keywords:** Alzheimer's disease, liraglutide, synapse damage, insulin receptors, PKA signaling, non‐human primates, diabetes, GLP‐1, tau pathology, neurodegeneration

## Abstract

Alzheimer's disease (AD) is a devastating neurological disorder that still lacks an effective treatment, and this has stimulated an intense pursuit of disease‐modifying therapeutics. Given the increasingly recognized link between AD and defective brain insulin signaling, we investigated the actions of liraglutide, a glucagon‐like peptide‐1 (GLP‐1) analog marketed for treatment of type 2 diabetes, in experimental models of AD. Insulin receptor pathology is an important feature of AD brains that impairs the neuroprotective actions of central insulin signaling. Here, we show that liraglutide prevented the loss of brain insulin receptors and synapses, and reversed memory impairment induced by AD‐linked amyloid‐β oligomers (AβOs) in mice. Using hippocampal neuronal cultures, we determined that the mechanism of neuroprotection by liraglutide involves activation of the PKA signaling pathway. Infusion of AβOs into the lateral cerebral ventricle of non‐human primates (NHPs) led to marked loss of insulin receptors and synapses in brain regions related to memory. Systemic treatment of NHPs with liraglutide provided partial protection, decreasing AD‐related insulin receptor, synaptic, and tau pathology in specific brain regions. Synapse damage and elimination are amongst the earliest known pathological changes and the best correlates of memory impairment in AD. The results illuminate mechanisms of neuroprotection by liraglutide, and indicate that GLP‐1 receptor activation may be harnessed to protect brain insulin receptors and synapses in AD. © 2018 The Authors. *The Journal of Pathology* published by John Wiley & Sons Ltd on behalf of Pathological Society of Great Britain and Ireland.

## Introduction

Alzheimer's disease (AD) is a neurodegenerative condition that shatters the lives of patients and family members, a situation that urgently demands the development of effective treatments. Recent evidence indicates that AD brains exhibit altered levels of components of the insulin signaling pathway and decreased responsiveness to insulin 1, 2, 3, 4, 5, 6. In particular, brain levels of insulin and insulin receptors (IRs) are reduced in AD patients and in experimental models of AD 5, 6, 7, 8, 9, 10. Such molecular links between deregulated insulin signaling in AD and diabetes have raised the prospect for novel therapeutic strategies based on anti‐diabetic agents 11, 12, 13, 14, 15.

Glucagon‐like peptide‐1 (GLP‐1) is a peptide hormone that increases insulin secretion and decreases glucagon secretion from the pancreas in a glucose‐dependent manner 16. GLP‐1 receptors (GLP‐1Rs) are present and functional in cultured neurons as well as in rodent and human brains 17, 18, 19, 20, 21, and GLP‐1R stimulation affects neuronal physiology, facilitating hippocampal synaptic plasticity, cognition, and cell survival 17, 22, 23. These studies demonstrate that infusion of GLP‐1 into the ventricles of the brain, or expression of the GLP‐1 receptor using an adeno‐associated virus vector, improves learning and memory of rats. Furthermore, other studies have shown that GLP‐1R analogs enhance synaptic plasticity in the brain 22, 24, 25, 26. Several additional studies demonstrate beneficial actions of GLP‐1R activation on memory in AD transgenic mice 5, 27, 28, 29, 30, 31, 32, which can be explained, at least in part, by the effects of GLP‐1R agonists decreasing AD‐related pathology. For example, the GLP‐1 receptor agonists exendin‐4 and liraglutide, a long‐lasting GLP‐1R agonist, exert many protective effects in AD pathology. Exendin‐4 or liraglutide reduces cortical and hippocampal plaque load and alleviates brain insulin resistance and glial activation in transgenic AD mice 5, 24, 28, 30, 33. Liraglutide lowers phosphorylated tau levels in cultured neurons and in mice and reduces hippocampal neuronal loss in transgenic AD mice 31, 32, 34, 35, 36. The studies in experimental models of the disease have led to clinical trials aimed at testing the efficacy of liraglutide in AD (ID: NCT01843075; ID: NCT01469351; ID: NCT02140983). Interestingly, liraglutide has been recently shown to improve cognition in individuals with mood disorders and Parkinson's disease 37, 38, 39, 40. However, little is known to date about the cellular mechanisms of action of GLP‐1R agonists in the brain. Some studies suggest that GLP‐1R agonists activate insulin‐related signaling pathways, including activation of PI3K 41, 42. Importantly, the molecular protective actions of liraglutide and other GLP‐1R agonists in the primate brain remain to be determined.

Here, we used a multi‐disciplinary strategy to investigate the beneficial effects of liraglutide in AD experimental models, ranging from cell culture to rodents and cynomolgus macaques (*Macaca fascicularis*). We show that liraglutide protects neurons from the deleterious actions of amyloid‐β oligomers (AβOs), proximal synaptotoxins in AD that accumulate in the brains of AD patients 43, 44 and impair insulin signaling 5, 7, 45, 46. Using cultured hippocampal neurons, we show that liraglutide decreases AβO‐induced synaptotoxicity and has protective actions on synapses, an effect mediated by protein kinase A (PKA) activation. In mice, we found that liraglutide prevents and reverses cognitive impairment and IR loss caused by intracerebroventricular (i.c.v.) administration of AβOs. Studies were extended to AβO‐injected non‐human primates (NHPs), an attractive model of AD we recently developed that presents key neuropathological correlates of human AD, including the presence of neurofibrillary tangles and synapse loss 47. We found that AβOs trigger a striking reduction in the levels of IRs in NHPs. While liraglutide fully blocked synapse and IR loss induced by AβOs *in vitro* and in mice, the drug was less effective in NHPs, but still afforded partial protection against loss of IRs and synapses and tau phosphorylation. NHP brains are highly similar to human brains, and studies using a validated NHP model of AD will likely be a key step towards understanding AD‐relevant pathogenic mechanisms. Furthermore, testing therapeutics in an NHP AD model may provide novel insight into mechanisms of action in the primate brain, thus enhancing the chances of success in translation to the human disease condition. Collectively, our results indicate that GLP‐1R agonists represent a promising approach that may help to prevent brain IR and synaptic pathology in AD.

## Materials and methods

Detailed information on the reagents and procedures is provided in the supplementary material, Supplementary materials and methods.

### Reagents

Aβ_1–42_ was from American Peptide (Sunnyvale, CA, USA). Ham's F12 media (HF12‐02) was from Caisson Labs (Smithfield, UT, USA). DMSO, bovine serum albumin (BSA), insulin, forskolin, 8‐bromoadenosine (8‐Br‐cAMP), exendin fragment 9‐39, and 3‐isobutyl‐1‐methylxanthine (IBMX) were from Sigma (St Louis, MO, USA). DAB was from DakoCytomation (Glostrup, Denmark). Prolong Gold Antifade, normal goat serum and DAPI were from Invitrogen (Carlsbad, CA, USA). Vectastain Elite ABC reagent was from Vector Laboratories Inc (Burlingame, CA, USA). Harris' hematoxylin and Entellan were from Merck (São Paulo, Brazil). In experiments *in vitro* and with Swiss mice, liraglutide was purchased from GL Biochem (Shanghai, China) and with NHPs, liraglutide was purchased from Novo Nordisk *(*Bagsværd, Denmark). PKI (14‐22) amide (myristoylated) and H89 were from Cell Signaling Technology (Danvers, MA, USA). ELISA kits for insulin and PKA activity were from Crystal Chem Inc (Elk Grove Village, IL, USA) and Enzo Life Sciences Inc (Farmingdale, NY, USA), respectively. All the antibodies used in this study are listed in the supplementary material, Tables S1 and S2.

### Aβ oligomers

Aβ oligomers (AβOs) were prepared from synthetic Aβ_1–42_ peptide as originally described 48. For experiments in NHPs, AβOs were prepared in cold Ham's‐F12. A complete description of the biophysical/biochemical characterization of the preparations of amyloid‐β oligomers used in the current work has been given in previous work from our group 27, 49, 50, 51. Oligomers were kept at 4°C and used up to 2 days after preparation to avoid fibrilization. The Aβ oligomer‐specific antibody (NU4) was generated and characterized in William L Klein's laboratory (Northwestern University, Evanston, IL, USA) 48.

### Mature hippocampal cultures and immunocytochemistry

Primary rat hippocampal neuronal cultures were prepared and developed according to previously described experimental procedures 7, 27, 52, 53. Cultures were used after 18–21 days *in vitro*. All procedures were approved by the Institutional Animal Care and Use Committee of the Federal University of Rio de Janeiro (protocol # IBQM 022) and are described in the supplementary material, Supplementary materials and methods. Cultures were exposed to 500 nm AβOs (or an equivalent volume of vehicle, containing 2% DMSO in PBS) for 3 h at 37°C. When present, insulin (1 μm), liraglutide (300 nm), forskolin (10 μm), 8‐Br‐AMPc (10 μm), PKI‐14‐22 (1 μm), and H89 (20 μm) were added to cultures 40 min before AβOs. When present, exendin 9‐39 (1 μm) was added 15 min before addition of liraglutide to cultures. After fixation with 4% paraformaldehyde for 15 min, cultures were permeabilized with 0.1% Triton X‐100 (Merck) for 5 min at room temperature, and non‐specific sites were blocked with 10% normal goat serum for 1 h before immunoreactions with anti‐synaptophysin or anti‐PSD‐95 specific antibodies. For immunoreaction with anti‐IRα, cultures were not permeabilized as described previously 7. After incubation with primary antibodies, cells were washed with PBS and incubated with secondary antibodies for 2 h at room temperature. The secondary antibodies used were AlexaFluor594‐conjugated goat anti‐rabbit IgG (1:2000) or AlexaFluor488‐conjugated goat anti‐mouse IgG (1:2000), both from Thermo Fisher Scientific (Waltham, MA, USA). Cultures were mounted with Prolong Gold Antifade with DAPI, and cells were imaged on a confocal Nikon C2 microscope.

### Intracerebroventricular injection of AβOs in mice and treatment with liraglutide

Male Swiss mice obtained from our animal facility were 2.5–3 months of age at the beginning of experiments. All procedures were approved by the Federal University of Rio de Janeiro Animal Care Committee (protocol number 134/15) and were in full compliance with the NIH Guide for Care and Use of Laboratory Animals. AβOs (10 pmol) or vehicle was injected in a final volume of 3 μl into the lateral ventricle of mice as previously described by our group 27, 49, 54, 55. Male Swiss mice received daily i.p. injections of liraglutide (25 nmol/kg) or vehicle (PBS) for 7 days.

### Intracerebroventricular injection of AβOs in NHPs and treatment with liraglutide

All macaques were maintained at the Centre for Neuroscience Studies at Queen's University (Kingston, Canada) under the close supervision of a lab animal technician and the Institute's veterinarian. All procedures were approved by the Queen's University Animal Care Committee and were in full compliance with the Canada Council on Animal Care (Animal Care Protocol Original Munoz, 2011‐039‐Or). Three sham‐operated NHPs, 9 (*n* = 1) and 16 years of age (*n* = 2), were used as controls and underwent the full surgical procedure but did not receive intracerebroventricular injections. After a recovery period of 2–4 weeks, six NHPs, 9 (*n* = 2) and 16 years old (*n* = 4), had a guide cannula inserted into the lateral ventricle and held in place with a custom‐designed grid system in the chamber. They then received intracerebroventricular (i.c.v.) injections of 10–100 μg of AβOs (∼1 injection per day every 3 days for up to 24 days) while sedated using ketamine (Vetoquinol, Québec, ON, Canada; 3 mg/kg), medetomidine (Sandoz, Québec, ON, Canada; 0.15 mg/kg), and glycopyrolate (Sandoz; 0.013 mg/kg). Two of these animals (16 years of age) received daily subcutaneous injections of liraglutide (0.006 mg/kg for the first week and 0.012mg/kg thereafter) beginning 1 week prior to AβO injections and continuing until the end of AβO injections. Termination and brain processing procedures may be found in the supplementary material, Supplementary materials and methods.

### ELISA

PKA and insulin ELISA assays were performed according to each manufacturer's instructions kit after optimization of sample dilution. Hippocampi from Swiss mice were collected in low protein‐adsorption plastic tubes and were either used fresh for ELISA or immediately frozen in liquid nitrogen, and stored at −80°C until analysis. Tissue samples analyzed for PKA activity were homogenized in buffer containing 20 mm Hepes, NP40 1%, 2 mm EDTA, 5 mm EGTA, 1 mm DTT (1,4‐dithiothreitol) with protease and phosphatase inhibitor cocktails to maintain PKA activity. All these reagents were from Sigma. Basal levels of PKA were evaluated without phosphodiesterase inhibitors. The tissue samples used for insulin detection were homogenized in buffer containing 10 mm Tris, 0.32 m sucrose, 1 mm EDTA, 1 mm EGTA, and 0.1% Triton, and supplemented with protease and phosphatase inhibitor cocktails. For insulin and PKA ELISA assays, mice were not subjected to fasting.

### Western blotting

Western immunoblot analysis of the macaque brains was performed as described previously 56. More details are provided in the supplementary material, Supplementary materials and methods.

### Image analysis

Twenty to thirty images were acquired from each region of interest (see supplementary material, Table S3) in each animal, with constant acquisition parameters. IRα and IRβ immunoreactivity intensities were determined using a multi‐threshold plug‐in within NIH ImageJ. For quantification of synaptic punctae, a total of 15–20 images per experimental condition were acquired. PSD‐95 (red) and synaptophysin (green) immunofluorescence was analyzed and quantified using the Puncta Analyzer plugin in NIH ImageJ as previously described 57. Researchers analyzing images were unaware of the experimental conditions. AT100‐ and CP13‐positive cells were analyzed in 20–30 z‐stack images from each region of interest for each animal.

### Electron microscopy

For ultrastructural analysis of synapses, samples from the prefrontal cortex of NHPs were fixed (4% paraformaldehyde) and post‐fixed by immersion in 1% osmium tetroxide in cacodylate buffer with 0.8% potassium ferrocyanide for 3 h at room temperature. Electron microscopy analysis was performed in masked fashion. Images were taken at 30 000× magnification. A total of 50 images were acquired per animal/experimental condition (*n* = 3 sham‐operated NHPs; *n* = 4 AβO‐injected NHPs; *n* = 2 liraglutide‐treated NHPs). Synaptic profiles were identified by the presence of the postsynaptic density and presynaptic vesicle. The cross‐sectional length of synaptic junctions was measured using NIH ImageJ.

### Experimental design and statistical analysis

Data are represented as averages ± SEM. All statistical analyses were performed using GraphPad Prism (version 6.0). One‐way ANOVA followed by the Bonferroni *post hoc* test was used, as indicated in the legends. Data from the novel object recognition task were analyzed by one‐sample *t*‐test, compared against a fixed value of 50% (as previously described 49). G^*^Power (G^*^Power version 3.1.9.2) was used to estimate the sample size required in cell culture and mice experiments. G*Power is a tool to compute statistical power analyses that allows determination of the sample size required to detect an effect of a given size with a given degree of confidence.

Specifically, in experiments using NHPs, in which ethical considerations in working with primates limited our ability to increase sample size, the numbers of NHPs are limited to three controls, four AβO‐injected NHPs, and two AβO‐injected NHPs that received liraglutide treatment. In this case, we have used G*Power after our results were obtained and based on the effect size observed in each analysis, the program has estimated the statistical power obtained according to the number of samples used in the experiments. Although the sample size was very limited, the statistical power (80%) allowed us to validate our conclusions in Figures 5 and 6 and supplementary material, Figures S3 and S4.

## Results

### Activation of cAMP/PKA signaling mediates protection by liraglutide against AβO‐induced synapse damage in hippocampal neurons

**Figure 1:**
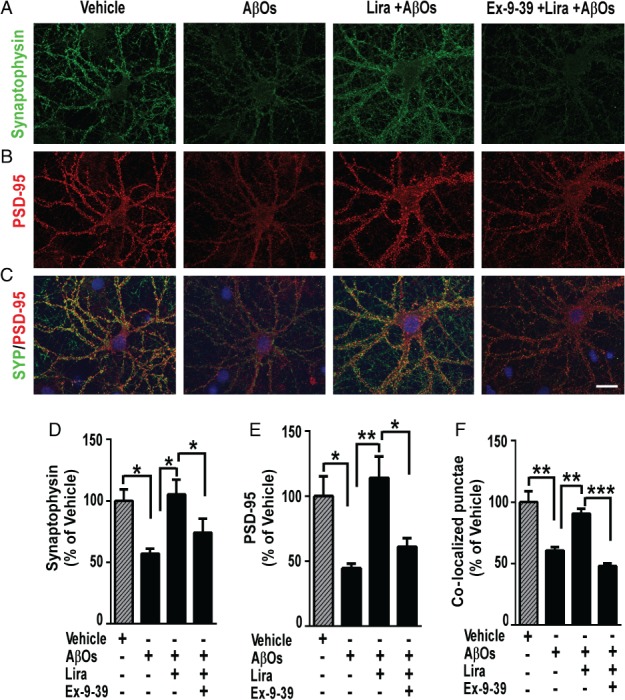
GLP‐1 receptor activation prevents AβO‐induced synapse loss in hippocampal neuronal cultures. (A–C) Representative images of cultured hippocampal neurons exposed to 500 nM AβOs or vehicle for 3 h and immunolabeled for synaptophysin (A; green) or PSD‐95 (B; red). Merged images (yellow) are shown in C. Where indicated, neurons were pre‐incubated for 40 min with liraglutide (300 nm), in the absence or presence of exendin (9‐39) (1 μm, added 15 min prior to liraglutide). Nuclear staining (DAPI) is shown in blue. Scale bar = 20 μm. (D–F) Integrated immunoreactivities for synaptophysin (D), PSD‐95 (E), and number of co‐localized synaptophysin/PSD‐95 punctae (F). Data are expressed as means ± SEM from three experiments from independent cultures (30 images analyzed per experimental condition per experiment). *p < 0.05; **p < 0.01; ***p < 0.001, compared with vehicle or AβOs; one‐way ANOVA followed by Bonferroni post hoc test. P value: In D: vehicle versus AβOs (p = 0.0416); AβOs versus Lira + AβOs (p = 0.0235); Lira + AβOs versus Ex‐9‐39‐Lira + AβOs (p = 0.0459). In E: vehicle versus AβOs (p = 0.0322); AβOs versus Lira + AβOs (p = 0.0097); Lira + AβOs versus Ex‐9‐39+Lira + AβOs (p = 0.0399). In F: vehicle versus AβOs (p = 0.0016); AβOs versus Lira + AβOs (p = 0.0087); Lira + AβOs versus Ex‐9‐39+Lira + AβOs (p = 0.0009).

Given recent evidence indicating that GLP‐1R activation is neuroprotective in cellular and mouse models of AD 5, 17, 22, 23, 25, 27, 28, 58, 59, we first sought to determine whether liraglutide might prevent the loss of synapses induced by AβOs in mature neuronal hippocampal cultures. Freshly prepared AβOs were routinely characterized by size exclusion chromatography, revealing a mixture of oligomers ranging from dimers, trimers, and tetramers to larger oligomers 49, 60. Exposure of neuronal cultures to AβOs (500 nm) for 3 h caused significant reductions in the levels of pre‐ and post‐synaptic markers, synaptophysin (Figure 1A, D) and PSD‐95 (Figure 1B, E), respectively, and in synapse density, estimated by the number of puncta with co‐localized synaptophysin/PSD‐95 labeling (Figure 1C, F). These measures of synapse damage were all attenuated by pretreatment of cultures with liraglutide (Figure 1). Control experiments showed that exendin (9‐39) amide, a potent GLP‐1R antagonist 61 and a competitive inhibitor of liraglutide, blocked the action of liraglutide (Figure 1), demonstrating that protection was specifically mediated by activation of GLP‐1Rs.

**Figure 2:**
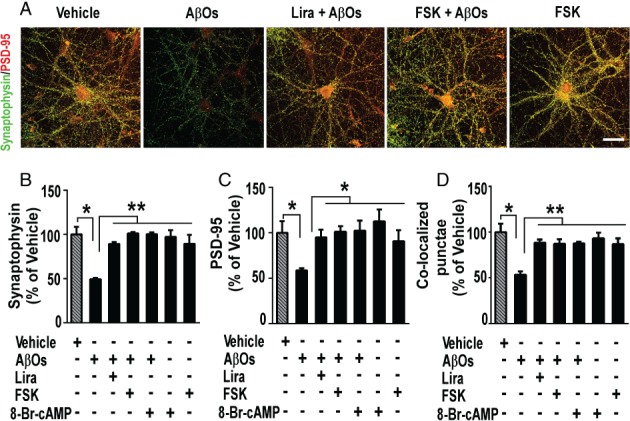
GLP‐1 receptor activation prevents AβO‐induced synapse loss via a cAMP‐dependent pathway. (A) Representative images of cultured hippocampal neurons exposed to 500 nm AβOs or vehicle for 3 h and immunolabeled for synaptophysin (green) and PSD‐95 (red). Where indicated, neurons were pre‐incubated with liraglutide (300 nm) or forskolin (FSK; 10 μm) for 40 min prior to addition of AβOs. Scale bar = 60 μm. (B–D) Integrated immunoreactivities for synaptophysin (B), PSD‐95 (C), and co‐localized synaptophysin/PSD‐95 punctae (D). Data are expressed as means ± SEM from four to six experiments from independent cultures (30 images analyzed per experimental condition per experiment). *p < 0.05; **p < 0.01; ***p < 0.001, compared with vehicle or AβOs; one‐way ANOVA followed by Bonferroni post hoc test. P value: In B: vehicle versus AβOs (p = 0.001); AβOs versus Lira + AβOs (p = 0.003); AβOs versus FSK + AβOs (p = 0.002); AβOs versus 8‐Br‐cAMP + AβOs (p = 0.001); AβOs versus FSK (p = 0.001); AβOs versus 8‐Br‐cAMP (p = 0.008). In C: vehicle versus AβOs (p = 0.0153); AβOs versus Lira + AβOs (p = 0.0412); AβOs versus FSK + AβOs (p = 0.0549); AβOs versus 8‐Br‐cAMP + AβOs (p = 0.0464); AβOs versus FSK (p = 0.04); AβOs versus 8‐Br‐cAMP (p = 0.003). In D: vehicle versus AβOs (p = 0.02); AβOs versus Lira + AβOs (p = 0.0012); AβOs versus FSK + AβOs (p = 0.0033); AβOs versus 8‐Br‐cAMP + AβOs (p = 0.0087); AβOs versus FSK (p = 0.0026); AβOs versus 8‐Br‐cAMP (p = 0.0017).

**Figure 3:**
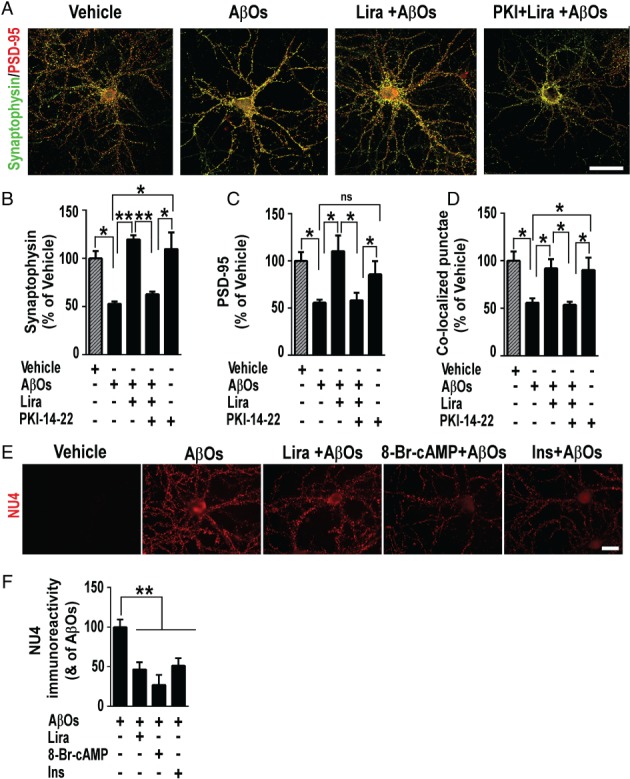
PKA activation is required for GLP‐1 receptor‐mediated prevention of AβO‐induced synapse loss. (A) Representative images of cultured hippocampal neurons exposed to 500 nm AβO or vehicle for 3 h and immunolabeled for synaptophysin (green) and PSD‐95 (red). Where indicated, neurons were pre‐incubated with liraglutide (300 nm) or PKI‐14,22 (1 μm) for 40 min. Additional results with H89 may be found in the supplementary material, Figure S1. Scale bar = 60 μm. (B–D) Integrated immunoreactivities for synaptophysin (B), PSD‐95 (C), and co‐localized synaptophysin/PSD‐95 punctae (D). Data are expressed as means ± SEM from three experiments from independent cultures (30 images analyzed per experimental condition per experiment). (E) Representative images from hippocampal neurons in culture exposed to 500 nm AβOs or vehicle for 3 h and immunolabeled for AβOs (NU4 antibody). Where indicated, cultures were pretreated with liraglutide (300 nm), 8‐Br‐cAMP (10 μm) or insulin (1 μm) for 15 min. Scale bar = 20 μm. (F) Integrated NU4 immunoreactivities. Data are expressed as means ± SEM from seven experiments from independent cultures (30 images analyzed per experimental condition per experiment). *p < 0.05; **p < 0.01; ***p < 0.001; ns (not significant); one‐way ANOVA followed by Bonferroni post hoc test. P value: In B: vehicle versus AβOs (p = 0.0137); AβOs versus Lira + AβOs (p = 0.0012); Lira + AβOs versus PKI + Lira + AβOs (p = 0.0038); PKI‐14‐22 versus PKI + Lira + AβOs (p = 0.0139); AβOs versus PKI‐14‐22 (p = 0.03). In C: vehicle versus AβOs (p = 0.05); AβOs versus Lira + AβOs (p = 0.02); Lira + AβOs versus PKI + Lira + AβOs (p = 0.02); PKI‐14‐22 versus PKI + Lira + AβOs (p = 0.06); AβOs versus PKI‐14‐22 (p = 0.08). In D: vehicle versus AβOs (p = 0.0158); AβOs versus Lira + AβOs (p = 0.04); Lira + AβOs versus PKI + Lira + AβOs (p = 0.03); PKI‐14‐22 versus PKI + Lira + AβOs (p = 0.02); AβOs versus PKI‐14‐22 (p = 0.04). In F: AβOs versus Lira + AβOs (p = 0.003); AβOs versus 8‐Br‐cAMP + AβOs (p = 0.0015); AβOs versus insulin + AβOs (p = 0.0097).

The mechanisms by which liraglutide exerts its beneficial actions in the brain remain largely unknown. Because GLP‐1R analogs have been reported to engage G_s_‐protein‐dependent signaling and PKA activation in peripheral tissues 16, 23, we next asked whether the cyclic AMP (cAMP)/PKA pathway, a ubiquitous cascade that modulates numerous cellular events within neurons 62, 63, underlies the protective actions of liraglutide on synapses. We first investigated whether boosting cAMP levels using forskolin, an adenylate cyclase activator, could attenuate the effect of AβOs on synapses. Interestingly, similar to liraglutide, forskolin prevented synapse loss induced by AβOs (Figure 2A–D). We further found that 8‐Br‐cAMP, a PKA activator resistant to degradation by phosphodiesterases, also prevented synapse damage induced by AβOs (Figure 2A–D). Next, we tested if H89, a non‐specific PKA inhibitor 64, 65, modulated the effects of liraglutide on synapses. In the presence of H89, liraglutide failed to prevent AβO‐induced synapse loss (supplementary material, Figure S1). Then we tested the effect of PKI‐14‐22, a specific competitive inhibitor of PKA 65, and showed that PKI‐14‐22 also blocked protection by liraglutide (Figure 3A–D), further indicating that protection of synapses is specifically mediated by PKA activation. These results collectively suggest that activation of the cAMP/PKA pathway underlies the neuroprotective actions of liraglutide.

Protection by insulin against AβO‐induced synapse damage has been shown to involve downregulation of AβO binding to neurons 7. Additional experiments thus aimed to determine whether liraglutide interferes with AβO‐neuronal binding. Consistent with our previous observations 7, insulin reduced AβO binding to hippocampal neurons (Figure 3E, F). Interestingly, liraglutide exhibited a similar effect, decreasing the binding of AβOs to synapses (Figure 3E, F), suggesting that this could be an additional mechanism of neuroprotection by liraglutide. The fact that 8‐Br‐cAMP also reduced AβO neuronal staining (Figure 3E, F) further underlines the notion that activation of cAMP/PKA signaling mediates neuroprotection against the toxic impact of AβOs.

### Liraglutide regulates PKA activity and protects against memory impairment induced by AβOs in mice

**Figure 4:**
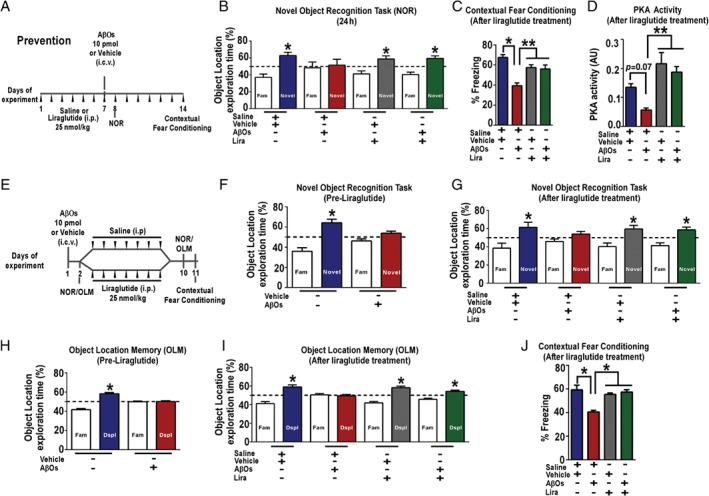
Liraglutide prevents and rescues AβO‐induced cognitive impairment in mice. (A) Experimental design of the treatment schedule with liraglutide (Prevention). Male Swiss mice received one daily injection of saline or liraglutide (25 nmol/kg) i.p. from days 1 to 7. On the last day of treatment with liraglutide, mice received an i.c.v. injection of AβOs (10 pmol) or vehicle. Twenty‐four hours after i.c.v. injection of AβOs (10 pmol) or vehicle, animals were trained in the novel object recognition (NOR) task. The percentage of time exploring familiar or novel objects in the test session is shown in B. Seven days after i.c.v. injection of AβOs (10 pmol) or vehicle, animals were trained in the contextual fear conditioning task. The percentage of freezing behavior in the test session is shown in C. (D) Basal PKA activity measured in the hippocampus of vehicle‐ or AβO‐injected mice treated with liraglutide (n = 6 mice per group). (E) Experimental design of an alternative treatment schedule with liraglutide (Reversal). Liraglutide (25 nmol/kg, i.p.) treatment began on day 3 and lasted for 7 days. Animals were trained and tested in the NOR before (F) and after (G) treatment with liraglutide. An independent group of mice was trained and tested in the object location memory (OLM) task before (H) and after (I) liraglutide treatment. (J) Eleven days after i.c.v. injection of AβOs (10 pmol) or vehicle and after treatment with liraglutide, animals were trained in the contextual fear conditioning task. The percentage of freezing behavior in the test session is shown. In B, C, F, and G–I, data are expressed as means ± SEM (n = 10–12 mice per group) of the percentage of time spent on each object presented at the test session and *p < 0.05, compared with a hypothetical value of 50% of exploration time; one‐sample Student's t‐test. P value: In B: p = 0.0095 (relative to vehicle), p = 0.8274 (relative to AβOs), p = 0.0389 (relative to vehicle + Lira), p = 0.0065 (relative to Lira + AβOs). In F: p = 0.0058 (relative to vehicle), p = 0.08 (relative to AβOs). In G: p = 0.04 (relative to vehicle), p = 0.1723 (relative to AβOs), p = 0.03 (relative to vehicle + Lira), p = 0.02 (relative to AβOs + Lira). In H: p = 0.002 (relative to vehicle), p = 0.8879 (relative to AβOs). In I: p = 0.005 (relative to vehicle), p = 0.54 (relative to AβOs), p = 0.001 (relative to vehicle + Lira), p = 0.002 (relative to AβOs + Lira). In C, D, and J, data are expressed as means ± SEM; *p < 0.05; **p < 0.01; ***p < 0.001; one‐way ANOVA followed by Bonferroni post hoc test. P value: In C: vehicle + saline versus AβOs + saline (p = 0.02); AβOs + saline versus vehicle + Lira (p = 0.03); AβOs versus Lira + AβOs (p = 0.002). In D: vehicle + saline versus AβOs + saline (p = 0.07); AβOs + saline versus vehicle + Lira (p = 0.001); AβOs versus Lira + AβOs (p = 0.002). In J: vehicle + saline versus AβOs + saline (p = 0.04); AβOs + saline versus vehicle + Lira (p = 0.03); AβOs versus Lira + AβOs (p = 0.02). Fam = familiar object used in the test session; Novel = novel object used in the test session; Dspl = displaced object used in the test session.

We next sought to determine whether liraglutide could prevent memory deficits induced by AβOs in mice. We have shown that intracerebroventricular (i.c.v.) injection of AβOs induces memory deficits and depressive‐like behavior in mice 49, 54. AβOs were freshly prepared before i.c.v. administration. Consistent with our previous reports, a single i.c.v. injection of AβOs (10 pmol) impaired declarative memory, assessed by the novel object recognition (NOR) task, 24 h post‐injection (Figure 4A, B). To assess whether liraglutide could prevent the AβO‐induced memory deficit, mice were systemically treated with liraglutide (25 nmol/kg, i.p.) for 7 days prior to AβO injection. As shown in Figure 4B, treatment with liraglutide prevented memory impairment caused by AβOs. We next evaluated whether liraglutide could prevent against AβO‐induced impairments in contextual fear conditioning memory. While AβO‐infused mice developed memory deficits in the contextual fear conditioning test, mice that received liraglutide treatment prior to AβOs presented freezing behavior similar to controls (Figure 4C). We next evaluated PKA activity in the hippocampi of these mice. Our results indicate that AβOs trigger a decrease in PKA activity in the mouse hippocampi, and liraglutide attenuated the effects of AβOs in PKA activity (Figure 4D).

We then evaluated whether liraglutide could rescue memory deficits induced by AβOs in mice. To this end, we initially confirmed memory impairment in mice that received an i.c.v. injection of AβOs (24 h post‐injection, Figure 4F). Mice were then separated into two groups, with one group treated with liraglutide and the other group treated with saline only (Figure 4E). NOR memory was tested again after 7 days of liraglutide treatment. Remarkably, while memory impairment persisted in the saline‐treated group of animals 9 days post‐injection of AβOs, AβO‐injected mice that were treated for 1 week with liraglutide showed normal performance in the NOR task (Figure 4G).

We further explored the protective role of liraglutide in a different group of mice tested in a hippocampal‐dependent version of the NOR memory task, the object location memory (OLM) test 66. As above, AβO‐induced memory impairment was assessed 24 h after AβO injection (Figure 4H) before mice were separated into two groups and i.p. treatment with liraglutide (or saline) was initiated (Figure 4E). As in the NOR task, OLM memory was impaired in AβO‐injected mice treated with saline 9 days post AβO injection, whereas treatment with liraglutide rescued memory in AβO‐injected mice (Figure 4I). Similar results were obtained when another group of mice was submitted to a contextual fear conditioning task, a hippocampus‐ and amygdala‐dependent test. When tested 24 days after the training session, AβO‐injected mice expressed significantly less freezing behavior compared with vehicle‐injected mice, whereas the performance of liraglutide‐treated AβO‐injected mice was similar to that of vehicle‐injected control animals (Figure 4J). Together, the results demonstrate that liraglutide is able to prevent and rescue the deleterious impact of AβOs on memory in mice.

### Liraglutide prevents AβO‐induced decrease in IRs in neuronal cultures and in mice

**Figure 5:**
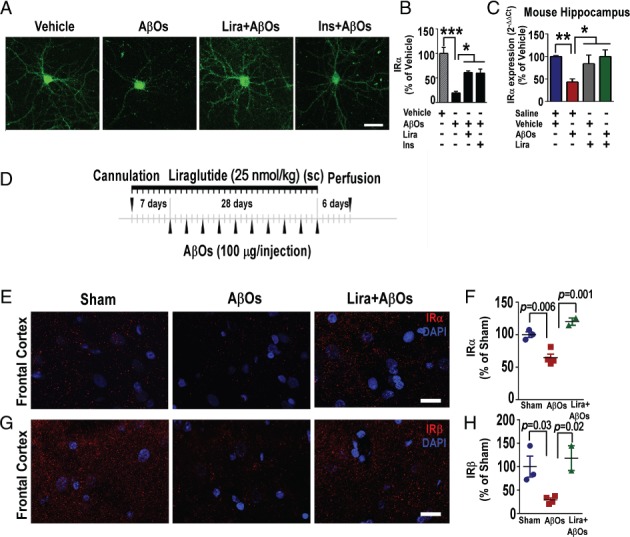
Liraglutide attenuates AβO‐induced loss of hippocampal insulin receptor in vitro, in mice and in the frontal cortex of non‐human primates (NHPs). (A) Representative images of cultured hippocampal neurons exposed to AβOs (500 nm) or vehicle for 3 h and immunolabeled for insulin receptor (IRα subunit). Scale bar = 60 μm. (B) Integrated immunoreactivity for IRα. Data are expressed as means ± SEM from three experiments from independent cultures (30 images analyzed per experimental condition per experiment). (C) Insulin receptor (IRα subunit) mRNA levels in the hippocampi of mice 10 days after i.c.v. injection of AβOs (10 pmol) or vehicle. Following AβO injection, animals were treated for 7 days with saline or liraglutide (25 nmol/kg; i.p.; n = 5 animals per group). **(**D) Experimental design for experiments with NHPs. Cannulated animals (see the Materials and methods section) received daily subcutaneous treatment with liraglutide (25 nmol/kg) 7 days before the first AβO injection. AβOs (100 μg) were administered i.c.v. every 3 days for 28 days, and animals were perfused 6 days after the last injection. (E, G) Representative images of IRα (E) and IRβ (G) immunolabeling in the frontal cortex of sham‐operated, AβO‐injected, or liraglutide‐treated AβO‐injected NHPs. Nuclear staining (DAPI) is shown in blue. Scale bar = 20 μm. (F, H) Integrated IRα (F) and IRβ (H) immunoreactivities (n = 3 sham‐operated, 4 AβO‐injected, and 2 liraglutide‐treated AβO‐injected NHPs). Results are expressed as means ± SEM; one‐way ANOVA followed by Bonferroni post hoc test. *p < 0.05; **p < 0.01; ***p < 0.001. P value: In B: vehicle versus AβOs (p = 0.0002); AβOs versus Lira + AβOs (p = 0.02); AβOs versus insulin (p = 0.0170). In C: vehicle + saline versus AβOs + saline (p = 0.005); AβOs + saline versus vehicle + Lira (p = 0.04); AβOs + saline versus Lira + AβOs (p = 0.03). In F: sham versus AβOs: p = 0.006; AβOs versus Lira + AβOs: p = 0.001. In H: sham versus AβOs: p = 0.03; AβOs versus Lira + AβOs: p = 0.02).

We and others have previously shown that IRs are removed from the neuronal surface in hippocampal cultures exposed to AβOs 7, 45. Since mounting evidence indicates that insulin signaling in the brain is crucial for mechanisms related to neuronal function and memory 67, 68, 69, 70, 71, we hypothesized that the beneficial actions of liraglutide on neurons could involve prevention of IR loss. We initially investigated this possibility using mature hippocampal neuronal cultures. We exposed cultures to AβOs (500 nm) for 3 h and evaluated the dendritic levels of IRαs. Liraglutide attenuated the decrease in IR levels in hippocampal neurons induced by AβOs (Figure 5A, B). Consistent with our previous reports, insulin prevented AβO‐induced IRα removal from dendrites (Figure 5A, B). It is interesting to note that liraglutide and insulin conferred similar protection against reduction in surface IRα levels in neurons exposed to AβOs.

We next examined *IRα*‐mRNA levels in the hippocampi of mice that received an i.c.v injection of AβOs and evaluated the effects of liraglutide on IR expression. Hippocampal levels of *IRα* mRNA were reduced by AβO injection in saline‐treated mice but were preserved in mice that started liraglutide treatment 24 h after AβOs (Figure 5C). This result indicates that liraglutide reverses the impact of AβOs on IRs in the hippocampi of mice. We next aimed to analyze insulin levels in the hippocampus of mice to detect possible alterations in the experimental groups used, and we did not observe alterations in insulin levels in the experimental conditions tested (supplementary material, Figure S2).

### AβO‐induced IR loss in NHPs is attenuated by liraglutide

In order to determine if the mechanisms of neuroprotection by liraglutide are similar in the primate brain, we conducted experiments using a recently developed NHP model of AD 47. In this model, we delivered i.c.v. injections of AβOs (∼100 μg every 3 days for 28 days) to adult cynomolgus macaques (*Macaca fascicularis*). We have demonstrated that AβOs diffuse throughout the NHP brain and that the model mimics several aspects of human AD pathology, including tau hyperphosphorylation and tangle formation, synapse loss, astrocytic/microglial activation, and impairment of IRS‐1 signaling 5, 47. Here, we extended our investigation of this model to evaluate the effects of AβOs on IRs and other memory‐related receptors, and to determine if liraglutide could protect neurons from those effects.

Decreased IR levels have been shown to be an important characteristic of AD brains 6, 8. We thus examined IR levels in the frontal cortex, hippocampus, and amygdala of four NHPs that received i.c.v. injections of AβOs and in three sham‐operated, control animals. Levels of both IRα and IRβ subunits were markedly reduced in the frontal cortex of NHPs that received i.c.v. AβO injections (Figure 5E–H and supplementary material, Figure S3A, B). AβOs further decreased the levels of IRα and IRβ in the hippocampus and amygdala (supplementary material, Figure S3C–F). Our previous work demonstrated that AβOs target neurons in these regions of NHP brains 47. These observations establish that AβOs trigger IR loss in the primate brain. GLP‐1Rs have been shown to be present in NHP brains 72, so we next investigated the effects of systemic liraglutide treatment in two NHPs that received AβO injections (Figure 5D). Daily liraglutide treatment started 1 week before AβO injections. In the frontal cortex of AβO‐injected NHPs, liraglutide treatment afforded protection against IRα and IRβ (Figure 5E–H and supplementary material, Figure S3A, B). Liraglutide further attenuated the decrease in IRα, but not that in IRβ, in the hippocampus (supplementary material, Figure S3C, D). On the other hand, no protective effect was observed in the amygdala of AβO‐injected NHPs (supplementary material, Figure S3E, F).

### Liraglutide alleviates AβO‐induced synapse loss in NHPs

**Figure 6:**
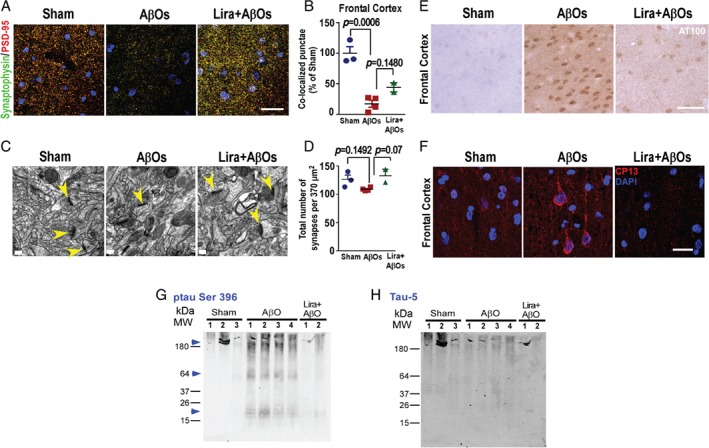
Liragutide attenuates AβO‐induced synapse damage and tau hyperphosphorylation in the brains of NHPs. (A) Representative images from the frontal cortex of sham‐operated, AβO‐injected, and liraglutide‐treated AβO‐injected NHPs immunolabeled for synaptophysin and PSD‐95 (merged images). Nuclear staining (DAPI) is shown in blue. Scale bar = 30 μm. (B) Number of co‐localized synaptophysin/PSD‐95 immunoreactive punctae per unit area (relative to sham‐operated NHPs) in the frontal cortex. Data are expressed as means ± SEM (n = 3 sham‐operated, n = 4 AβO‐injected NHPs, n = 2 liraglutide‐treated AβO‐injected NHPs). Representative images from synaptophysin and PSD‐95 may be found in the supplementary material, Figure S5. (C) Representative electron micrographs from the frontal cortex of sham‐operated, AβO‐injected, and liraglutide‐treated AβO‐injected NHPs. Yellow arrowheads indicate structures identified as synapses (presence of post‐synaptic density as opposed to a presynaptic specialization). (D) Quantification of synapse density in electron micrograph images. Data are expressed as means ± SEM (n = 3 sham‐operated, n = 4 AβO‐injected NHPs, n = 2 liraglutide‐treated AβO‐injected NHPs). (E, F) Representative images of the frontal cortex of sham‐operated, AβO‐injected, and liraglutide‐treated AβO‐injected NHPs (as indicated) immunolabeled for (E) AT100 (scale bar = 50 μm) or (F) CP13 (scale bar = 20 μm). For CP13, z‐stack projections were performed. Nuclear staining (DAPI) is shown in blue. (G) Western blot probed with anti‐tau pSer 396 revealed enhanced tau phosphorylation (64 kDa) in all AβO‐injected NHPs compared with control brains (blue arrowheads) (previously shown by Forny‐Germano et al 47). Liraglutide‐treated AβO‐injected NHPs revealed decreased tau pSer 396 in the two NHPs treated with liraglutide (see blue arrowhead). Note the presence of high‐molecular‐mass phospho‐tau‐reactive bands (> 150 kDa) in brain extracts from the frontal cortex of AβO‐injected NHPs (blue arrowhead). In addition, note the low‐molecular‐mass phosphorylated tau fragments (< 20 kDa) which were observed only in brain extracts from AβO‐injected macaques (see blue arrowhead as indicated). (H) A similar labeling profile between control and AβO‐injected NHPs was observed when the same membrane was probed with the anti‐Tau5 antibody. Data are expressed as means ± SEM; one‐way ANOVA followed by Bonferroni post hoc test. P value: In B: sham versus AβOs: p = 0.0006; AβOs versus Lira + AβOs: p = 0.1480. In D: sham versus AβOs: p = 0.1492; AβOs versus Lira + AβOs: p = 0.0796.

Synapse loss is the best pathological correlate of the severity of dementia in AD 73, 74, 75. Our previous study demonstrated that AβOs induced loss of synaptophysin and PSD‐95 in different NHP brain regions 47. Here, we assessed whether AβOs induced a decrease in synapse density, evaluated by co‐localization between synaptophysin and PSD‐95 immunoreactivities, and also tested if liraglutide could protect against AβO‐induced synapse loss. We found decreased densities of synapses in the frontal cortex, hippocampus, and amygdala of NHPs receiving AβOs compared with sham‐operated animals (Figure 6A–D and supplementary material, Figures S4 and S5). Liraglutide conferred modest protection against the loss of synaptophysin (supplementary material, Figures S4A–C and S5A, C), PSD‐95 (supplementary material, Figures S4D–F and S5B, D), and density of synapses (Figure 6A, B and supplementary material, Figures S4G–I and S5E) triggered by AβOs in the hippocampus, frontal cortex, and amygdala. As demonstrated in our previous study 47, electron microscopy (EM) analysis revealed that AβOs induced a decrease in synapse number in the frontal cortex of NHPs (Figure 6C, D). Liraglutide treatment attenuated the structural loss of synapses in the brains of AβO‐injected NHPs (Figure 6C, D).

### Liraglutide attenuates AβO‐induced tau hyperphosphorylation in NHPs

We next aimed to determine if liraglutide treatment could attenuate AβO‐induced AD‐like tau phosphorylation in the brains of NHPs. We evaluated abnormal tau phosphorylation in our model using the AT100 antibody, which recognizes tau phosphorylated at serine residue 212 and threonine residue 214 76, and the CP13 antibody, which recognizes tau phosphorylated at serine residue 202 77. Similar to data presented in our recent study 47, we observed increases in AT100‐ and CP13‐positive neurons (Figure 6E, F and supplementary material Figure S6). Interestingly, fewer AT100‐ and CP13‐positive neurons were observed in NHPs treated with liraglutide (Figure 6E, F). We next performed phospho‐tau immunoblots of frontal cortex extracts from all the NHPs used in this study. In accordance with our previous findings 47, western blots revealed enhanced tau phosphorylation (∼64 kDa) in the four AβO‐injected NHPs compared with sham‐operated controls (Figure 6G). We further noted the presence of high molecular mass phospho‐tau‐reactive bands (> 180 kDa) in AβO‐injected NHPs (Figure 6G). Such aggregates may correspond to tau oligomers, increasingly thought to be relevant for AD‐related neurodegeneration 78, 79, 80, 81, 82, 83. In addition, a low molecular mass (∼20 kDa) phosphorylated tau fragment was observed only in brain extracts from AβO‐injected NHPs. Interestingly, the two liraglutide‐treated NHPs presented decreases in phospho‐tau levels. When the same membrane was probed with the anti‐Tau5 antibody (which recognizes both phosphorylated and non‐phosphorylated isoforms of tau), we observed similar labeling profiles in the brain extracts from all NHPs (Figure 6H). Collectively, the results indicate that liraglutide decreases abnormal tau phosphorylation in the primate brain.

## Discussion

We have used a multi‐disciplinary approach to investigate the protective actions of liraglutide in different experimental models of AD, ranging from hippocampal cell cultures to mice to NHPs. Specifically, we aimed to determine the beneficial effects of liraglutide against AβOs, considered key synaptotoxins in AD 43, 44, 48, 84. Collectively, we found that liraglutide exerts protective actions on synapses through a mechanism that involves cAMP/PKA activation. Liraglutide reversed cognitive impairment and IR loss caused by AβOs in mice, and the results provided initial evidence that it also exerts partial neuroprotective actions in NHPs.

AβOs are small, diffusible aggregates of the Aβ peptide that accumulates in AD brains 43, 85 and have been shown to cause defective neuronal insulin signaling 5, 7, 45. Studies using cell cultures and rodent models have shown that AβOs induce AD‐like pathology, including neuronal tau hyperphosphorylation 52, oxidative stress 60, and inhibition of synaptic plasticity and memory 44, 85. An interesting recent study demonstrated that the decreased insulin signaling caused by AβOs induces neuronal cell cycle re‐entry, leading to neuron death 46. We showed that AβOs cause AD‐like pathology in NHPs, including tau hyperphosphorylation, tangle formation, brain inflammation, and synapse loss 47, and here we extended the characterization of this NHP model of AD to follow the impact of AβOs on IRs, characteristically affected in AD patients. NHP models of AD such as ours may help to bridge the gap between promising rodent research and the human disease condition, as the NHP brain shares many similarities with the human brain.

Data from neuropathology and genetic studies, as well as studies using rodent models of AD, have built a strong body of evidence supporting the notion that Aβ oligomerization and accumulation are key events leading to brain dysfunction and memory loss in AD 85. Previous pre‐clinical studies of Aβ immunotherapy in AD transgenic mouse models and aged NHPs have provided valuable information on the therapeutic effect in animal models 86, 87. It was recently reported that aducanumab, a monoclonal antibody against Aβ, decreases Aβ plaque density and slows cognitive decline in AD patients 88. That study reinforced the connection between Aβ and cognition. However, translation of immunotherapy into Alzheimer's patients was found to be challenging 89, indicating that we still need to better understand the mechanisms underlying AD. Identifying the cellular and molecular impacts of AβOs in the brains of NHPs will contribute to understanding the pathways leading to neurodegeneration and dementia, and has the potential to contribute to the development of effective strategies to prevent and/or treat AD.

In our study, the protective effects of liraglutide on synapses were found to involve regulation of PKA activity. According to our findings, previous studies have demonstrated that Aβ leads to impairment of the cAMP/PKA/CREB pathway 90, 91. These findings are consistent with some reports showing decreased PKA activity in AD brain 92, 93, 94. On the other hand, a recent study did not observe decreases in PKA levels in the prefrontal cortex of AD patients 95. In addition, a very recent study showed that insulin deficiency induces the activity of PKA and results in tau phosphorylation 96. This may suggest that the impairments in the cAMP/CREB pathway might not be solely attributed to PKA activity. Nonetheless, while these studies collectively indicate that PKA activity seems to be deregulated in AD and that fine regulation of its activity is important for neuronal function, further studies investigating PKA activity in AD are anticipated.

Here, we used hippocampal cultures and mice treated with liraglutide for extensive molecular and behavioral testing before moving to the more complex study with NHPs to follow brain pathology. The opportunity arose to test the effects of liraglutide in two NHPs. We acknowledge that the number of animals treated with liraglutide is low. Nonetheless, the data obtained from the two NHPs that received liraglutide treatment were quite consistent and offer initial insight into the effects of GLP‐1R agonists in the primate brain. We found that while liraglutide fully protected the brains of mice from AβO‐induced cognitive impairment and reversed cognitive damage and loss of *IR* mRNA content, the drug conferred partial protection against receptor and synapse loss in specific brain regions analyzed in NHPs. Methodological issues might explain the modest effect of liraglutide in some brain regions of NHPs, including the dose of liraglutide or a treatment period that was insufficient to lead to a more robust protective effect. It is also possible that due to the higher complexity of the primate brain, it might be challenging to heal it when some key processes are impacted. We further note that while several studies using cultures and rodent models of AD have demonstrated a robust impact on synapses 97, 98, 99, 100, studies in human AD brains reveal smaller decreases in synapses by EM analysis, ranging from 15% to 38% fewer synapses 73, 101, 102, 103. In our study, we found that the synapse number was decreased by 15% in AβO‐injected NHPs, compared with controls, which is consistent with some observations from human AD tissue. Nonetheless, it is also possible that the young age of the NHPs used in our study (middle‐aged) protected the animals against a more pronounced effect on synaptic pathology.

We demonstrated here that IR pathology is triggered by AβOs in NHPs. This finding supports a growing body of evidence showing that impaired IR signaling is a key AD feature 2, 5, 8, 12, 13. It further suggests that positive effects of liraglutide on neuronal insulin signaling involve preservation of IRs at the neuronal plasma membrane. Studies using *in vitro* and *in vivo* experimental models indicate that insulin signaling in the brain plays key roles in proper neuronal function, regulates neuronal survival, acts as a growth factor, and promotes healthy circuit function and plasticity 67, 68, 104, 105. Stimulation of insulin signaling is being considered an attractive strategy to preserve memory decline in AD 12, 13, 106, 107, 108. Intranasal insulin treatment improves memory in healthy adults, without changing blood levels of insulin or glucose 13, 106, 107, 109, 110, in agreement with the proposed role of insulin signaling in brain regions associated with cognition 70, 105. Moreover, intranasal insulin enhances verbal memory in memory‐impaired subjects 106 and, importantly, improves performance in early AD patients 106, 108. A large clinical trial is under way to test whether different types of insulin improve memory when administered intranasally to patients with mild cognitive impairment or early AD (ID: NCT01767909). Insight into the molecular mechanisms underlying the neuroprotective actions of insulin in AD came from studies showing that insulin protects neurons from AβOs 7, 111 and has beneficial effects in transgenic AD mice 112, 113. Approaches to increase IR levels may thus be therapeutically relevant.

There is significant interest in the use of GLP‐1R agonists as therapeutic agents in neurodegenerative diseases, particularly in Alzheimer's and Parkinson's diseases, and in mood disorders 4, 114. In peripheral tissue, GLP‐1R stimulation has insulinotropic actions and restores glucose homeostasis 16, 115, and several GLP‐1 analogs are currently used for diabetes treatment 16, 116. We recently demonstrated that the GLP‐1R agonist exendin‐4 suppresses IRS‐1 inhibition in APP/PS1 transgenic mice 5 and that liraglutide alleviates the PKR/eIF2α‐P pathway both in APP/PS1 mice and in the same NHPs used in the current study 27. Taken together, the current results demonstrate a neuroprotective role of liraglutide in several models of AD, and the protective effects on IRs suggest that a combined approach using liraglutide to prevent IR downregulation along with insulin might be an attractive therapeutic approach.

Currently, there is no effective treatment for AD and there is an intense pursuit of novel disease‐modifying therapeutics. In humans, disruption of brain connectivity is thought to develop well before evident brain pathology and dementia. Synapse loss has been proposed as the best neuropathological correlate of the extent of dementia in AD 73, 74, 75. This is consistent with early deficiencies in synapses and memory impairment in AD mouse models 49, 97, and with the alterations of synapses in NHPs that we report here and in our previous study 47. Therefore, new treatments that intervene at early stages of AD by strengthening synapses present a promising approach to tackle an unmet medical need. Testing therapeutics in NHPs will likely be a key step to develop a successful pharmacological strategy for AD, as human and NHP brains share considerable similarities in terms of overall architecture and organization of functional networks 87. A very recent study reported that AD patients treated with liraglutide were spared from cerebral glucose metabolism impairments over 6 months 117. This was a small trial and it is unknown whether there was a concomitant decrease in AD‐related pathology, but larger ongoing trials may provide such information in coming years. The results presented here collectively indicate that a GLP1‐R agonist presents beneficial actions in the mouse and NHP brain and, along with recent clinical data, suggest that GLP1‐R activation may represent a promising new approach to help prevent abnormal tau phosphorylation, as well as IR and synapse loss, thereby contributing to protect the brains of AD patients.

## Author contributions statement

AFB, LF‐G, JRC, NL, JBM, SEB, BCC, AL, DB, JF, SM, and MDG performed the experiments. AFB, LF‐G, JRC, NL, JBM, SEB, BCC, AL, DB, JF, SM, AMB, J‐CH, FDF, DPM, and STF analyzed and/or discussed data. DPM and FDF designed experiments in NHPs, and DPM performed experiments in NHPs. WLK and CH contributed reagents, materials, and analysis tools. FDF, DPM, and STF supervised the project. FDF, DPM, STF, AFB, JRC, and SEB wrote the manuscript.

**SUPPLEMENTARY MATERIAL ONLINE**

**Supplementary materials and methods**

**Supplementary figure legends**

**Figure S1.** PKA activation is required for GLP‐1 receptor‐mediated prevention of AβO‐induced synapse loss

**Figure S2.** Intracerebroventricular AβO injection or liraglutide treatment does not interfere with hippocampal insulin levels in mice

**Figure S3.** Effects of liraglutide on AβO‐induced loss of insulin receptor in the frontal cortex, hippocampus, and amygdala of NHPs

**Figure S4.** Liragutide attenuates AβO‐induced synapse damage in the hippocampus and amygdala of NHPs

**Figure S5.** Representative images of synapse densities in the frontal cortex and amygdala of NHPs

**Figure S6.** Representative images of hippocampus and amygdala immunolabeled for AT100 or CP13 in the NHPs

**Table S1.** List of the primary antibodies used

**Table S2.** List of the secondary antibodies used

**Table S3.** Nomenclature for specific brain regions analyzed following intracerebroventricular injections of AβOs in NHPs

## Supporting information


**Supplementary materials and methods**

**Supplementary figure legends**

**Figure S1.** **PKA activation is required for GLP‐1 receptor‐mediated prevention of AβO‐induced synapse loss. (A)** Representative images of cultured hippocampal neurons exposed to 500 nm AβOs (or vehicle) for 3 h and immunolabeled for synaptophysin (green)/PSD‐95 (red). Where indicated, neurons were pre‐incubated with liraglutide (300 nm) or H‐89 (10 μm) for 40 min. Scale bar = 60 μm. Insets show higher‐magnification images of selected dendrite segments. Scale bar = 10 μm. Integrated immunoreactivities for synaptophysin **(B)**, PSD‐95 **(C)** or co‐localized synaptophysin/PSD‐95 puncta **(D)**. Data are expressed as means ± SEM from three experiments from independent neuronal cultures (30 images analyzed per experimental condition per experiment). *p < 0.05, one‐way ANOVA followed by Bonferroni post hoc test. P value: In **B**: vehicle versus AβOs (p = 0.0073); AβOs versus Lira + AβOs (p = 0.0012); Lira + AβOs versus H89 + Lira + AβOs (p = 0.023); H89 versus Lira + AβOs (0.01); AβOs versus H89 (p = 0.04). In **C**: vehicle versus AβOs (p = 0.0006); AβOs versus Lira + AβOs (p = 0.0074); Lira + AβOs versus H89 + Lira + AβOs (p = 0.0014); H89 versus Lira + AβOs (0.0239); AβOs versus H89 (p = 0.051). In **D**: vehicle versus AβOs (p = 0.0009); AβOs versus Lira + AβOs (p = 0.012); Lira + AβOs versus H89 + Lira + AβOs (p = 0.03); H89 versus Lira + AβOs (0.0006); AβOs versus H89 (p = 0.045).

**Figure S2.** **Intracerebroventricular AβO injection or liraglutide treatment does not interfere with hippocampal insulin levels in mice.** Hippocampal insulin levels were measured 9 days after i.c.v. injection of AβOs (10 pmol) or vehicle. Prior to AβO injection, animals were pretreated for 7 days with saline or liraglutide (25 nmol/kg; i.p.; n = 5–6 animals per group). One‐way ANOVA followed by Bonferroni post hoc test. P value: vehicle + saline versus AβO + saline (p = 0.8036); AβOs + saline versus vehicle + Lira (p = 0.8834); AβOs + saline versus Lira + AβOs (p > 0.9999).

**Figure S3.** **Effects of liraglutide on AβO‐induced loss of insulin receptor in the frontal cortex, hippocampus, and amygdala of NHPs.** Representative images of frontal cortex, hippocampus, and amygdala of sham‐operated, AβO‐injected or liraglutide‐treated AβO‐injected NHPs (as indicated) immunolabeled for IRα (A, E, I) or IRβ (C, G, K). Scale bar = 50 μm. Quantification of integrated optical density for immunoreactivities of IRα or IRβ in the frontal cortex (B and D, respectively), hippocampus (F and H, respectively), and amygdala (J and L, respectively). Data are expressed as means ± SEM; n = 3 sham‐operated, n = 4 AβO‐injected, n = 2 liraglutide‐treated AβO‐injected NHPs. One‐way ANOVA followed by Bonferroni post hoc test. P value: In B: sham versus AβOs: p = 0.001; AβOs versus Lira + AβOs: p = 0.02. In D: sham versus AβOs: p = 0.01; AβOs versus Lira + AβOs: p = 0.14. In F: sham versus AβOs: p = 0.02; AβOs versus Lira + AβOs: p = 0.007. In H: sham versus AβOs: p = 0.002; AβOs versus Lira + AβOs: p = 0.97. In J: sham versus AβOs: p = 0.0432; AβOs versus Lira + AβOs: p = 0.99. In L: sham versus AβOs: p = 0.004; AβOs versus Lira + AβOs: p = 0.44.

**Figure S4.** **Liragutide attenuates AβO‐induced synapse damage in the hippocampus and amygdala of NHPs.** Representative images from the dentate gyrus of sham‐operated, AβO‐injected or liraglutide‐treated AβO‐injected NHPs (as indicated) immunolabeled for synaptophysin (**A**) or PSD‐95 (**D**). Merged images are shown in (**G**). Nuclear staining (DAPI) is shown in blue. Scale bar = 30 μm. Graphs represent the number of punctae per unit area (relative to sham‐operated NHPs) for synaptophysin (**B, C**) or PSD‐95 (**E, F**) in different brains regions (as indicated). For representative images from amygdala see **Figure S5. (H, I)** Number of co‐localized synaptophysin/PSD‐95 immunoreactive punctae per unit area (relative to sham‐operated NHPs). Data are expressed as means ± SEM. (n = 3 sham‐operated, n = 4 AβO‐injected NHPs, n = 2 liraglutide‐treated AβO‐injected NHPs). Data are expressed as means ± SEM. One‐way ANOVA followed by Bonferroni post hoc test. P value: In **B**: sham versus AβOs: p = 0.0009; AβOs versus Lira + AβOs: p = 0.09. In **C**: sham versus AβOs: p = 0.001; AβOs versus Lira + AβOs: p = 0.0049. In **E**: sham versus AβOs: p = 0.0003; AβOs versus Lira + AβOs: p = 0.03. In **F**: sham versus AβOs: p = 0.0003; AβOs versus Lira + AβOs: p = 0.0251. In **H**: sham versus AβOs: p = 0.0001; AβOs versus Lira + AβOs: p = 0.0042. In **I**: sham versus AβOs: p = 0.0001; AβOs versus Lira + AβOs: p = 0.002.

**Figure S5.** **Representative images of synapse densities in the frontal cortex and amygdala of NHPs.** Representative images from the frontal cortex or amygdala of sham‐operated, AβO‐injected or liraglutide‐treated AβO‐injected NHPs immunolabeled for synaptophysin (green) **(A, C)** and PSD‐95 **(B, D)** (red). Merged images from amygdala areas shown in **E**. Scale bar = 30 μm

**Figure S6.** **Representative images of hippocampus and amygdala immunolabeled for AT100 or CP13 in the NHPs.** Representative images of dentate gyrus and amygdala of sham‐operated, AβO‐injected or liraglutide‐treated AβO‐injected NHPs (as indicated) immunolabeled for AT100 **(A)** or CP13 **(B)**. Scale bar = 50 μm in **A** and 20 μm in **B**. For CP13, z‐stack projections were performed. Nuclear staining (DAPI) is shown in blue.

**Table S1.** List of the primary antibodies used

**Table S2.** List of the secondary antibodies used

**Table S3.** Nomenclature for specific brain regions analyzed following intracerebroventricular injections of AβOs in NHPs
