# Supplementary material for: The diabetes drug liraglutide reverses cognitive impairment in mice and attenuates insulin receptor and synaptic pathology in a non‐human primate model of Alzheimer's disease
Source: J Pathol. 2018 Apr 2;245(1):85–100. doi: 10.1002/path.5056 (PMC5947670; doi:10.1002/path.5056)
Supplement: Supplementary file 1 — Supplementary materials and methods [file PATH-245-85-s001.doc]

**Supplementary materials and methods**

Reference numbers refer to the main text reference list

**Animals**

Male Swiss mice obtained from our animal facility were 2.5–3 months of age at the beginning of experiments. All procedures were approved by the Federal University of Rio de Janeiro Animal Care Committee (protocol number 134/15) and were in full compliance with the NIH Guide for Care and Use of Laboratory Animals. All macaques were maintained at the Centre for Neuroscience Studies at Queen’s University (Kingston, Canada) under the close supervision of lab animal technicians and the Institute’s veterinarian. All procedures were approved by the Queen’s University Animal Care Committee and were in full compliance with regulations of the Canada Council on Animal Care (Animal Care Protocol Original Munoz, 2011-039-Or). Mice were housed in groups of five in each cage with free access to food and water, with controlled room temperature and humidity, and under a 12 h light/dark cycle. For i.c.v. injection of AβOs, animals were anesthetized for 7 min with 2.5% isoflurane (Cristália, São Paulo, Brazil) using a vaporizer system and gently restrained only during the injection procedure itself, as described. A 2.5 mm long needle was unilaterally inserted 1 mm to the right of the midline point equidistant from each eye and 1 mm posterior to a line drawn through the anterior base of the eye [27,49,54,55,99]. AβOs (10 pmol) or vehicle were injected in a final volume of 3 µl, and the needle was kept in place for 30 s to avoid backflow. Mice that showed signs of misplaced injections or any sign of hemorrhage (5% of cases, on average) were excluded from statistical analysis.

Nine female cynomolgus macaques (*Macaca fascicularis*, body weights 4.7–7.0 kg) were used. Animals (three sham-operated animals, four AβO-injected animals, and two liraglutide-treated AβO-injected animals) underwent a single surgical procedure to anchor a dental acrylic explant to the skull with Teflon or titanium screws. The explant included a chamber over a small midline craniotomy to access the lateral ventricles. Anesthesia was induced by ketamine (Vetoquinol, Québec, ON, Canada; 10–15 mg/kg, intramuscular) and diazepam (Sandoz, Québec, ON, Canada; 0.25–0.5 mg/kg, intramuscular). Animals were given glycopyrrolate (Sandoz; 0.013 mg/kg) and were intubated. During surgery, anesthesia was maintained with isoflurane (Fresenius Kabi, Toronto, ON, Canada; 1–3%), while meloxicam (Merck, Québec, ON, Canada; 0.2 mg/kg) and cefazolin (Sandoz; 22 mg/kg) were also administered. Correct placement of the chamber was assessed by MRI. Even though the experimental design involved injection of a fixed amount of 100 µg of AβOs per injection in all NHPs, it is important to note that in practice this amount was somewhat variable amongst animals, due to procedural limitations at the moment of injection (including partial clogging of the cannulas and liquid reflux through the cannula following injection). Approximately 1 week after completion of the experimental protocol, NHPs were sedated with intramuscular ketamine (10 mg/kg), followed by intravenous sodium pentobarbital (25 mg/kg) and heparin (Fresenius Kabi, Toronto, ON, Canada). They were then perfused intracardially with PBS followed by 4% paraformaldehyde in PBS. Brains were removed and stored in 4% paraformaldehyde, and then processed for neurohistology by NeuroScience Associates (Knoxville, TN, USA) into coronal 40-µm-thick sections.

**Immunocytochemistry analysis**

A total of 15–30 images per experimental condition were acquired. PSD-95 (red), synaptophysin (green), and synapses (defined as a juxtaposition between synaptophysin and PSD95 puncta) were analyzed and quantified using the Puncta Analyzer plugin in NIH ImageJ as previously described [57,97,98,99]. For IRα, cells were fixed and blocked as described above and were double-labeled with oligomer-specific NU4 mouse monoclonal antibody plus IRα rabbit polyclonal antibody followed by Alexa-conjugated secondary antibodies (Invitrogen, CA, USA).

Analyses were performed in a blinded fashion. Experiments were performed at least in triplicate and graphs represent means of all experiments

**Novel object recognition (NOR) and object location memory (OLM) tasks**

NOR and OLM were carried out in an open field arena measuring 0.3 (*w*) × 0.3 (*d*) × 0.45 (*h*) m. Test objects were made of glass or plastic and had different shapes, colors, sizes, and textures. During behavioral sessions, objects were fixed with tape to the floor of the arena so the animals could not move them. None of the objects used in our experiments evoked innate preference. Before training, each animal was submitted to a 5 min habituation session, in which they were allowed to freely explore the empty arena. During habituation sessions, the number of lines that each animal crossed on the floor (number of crossings) and the number of rearings (elevation on rear paws, denoting exploratory behavior) were recorded to verify possible effects of treatments on locomotor and exploratory activities. No differences were seen in the exploratory or locomotor behavior of mice after any of the treatments. Training consisted of a 5 min session during which animals were placed at the center of the arena in the presence of two identical objects positioned in a straight line. The amount of time spent exploring each object was recorded. Sniffing and touching the object were considered as exploratory behavior. The arena and objects were cleaned thoroughly between trials with 40% ethanol to eliminate olfactory cues. Two hours after training, animals were again placed in the arena for the test session.

In the NOR test session, one of the two objects used in the training session was replaced by a new one, whereas in the OLM test session, one of the objects was moved diagonally in the arena. Again, the amount of time exploring either the familiar or the novel/displaced object was measured by a trained researcher blinded to the identity of the experimental groups. Results are expressed as percentage of time exploring each object during the training or test session, and were analyzed using a one-sample Student’s *t*-test comparing the mean exploration time for each object with the fixed value of 50%. By definition, animals that recognize the familiar object as such (i.e. normal learning) explore the novel/displaced object for significantly longer than 50% of the total time of exploration.

**Contextual fear conditioning**

The conditioning chamber (25 × 25 × 25 cm) was built of aluminum walls, with a methacrylate door and a grid floor composed of 1-cm-spaced stainless steel bars connected to a shock generator (Panlab®, Harvard Apparatus, Cornellà, Spain). In the training session, mice were allowed to freely explore the conditioning box for 3 min and then received two footshocks (0.35 mA shock for 2 s, with a 30 s interval). Animals were allowed to remain in the chamber for 30 s after the last shock and then returned to their home cages. After 24 h, animals were again placed in the conditioning chamber for 5 min. Freezing behavior was automatically quantified by the Freezing® software version 1.3.04 (Panlab).

**Immunohistochemistry**

Immunohistochemistry was performed on 40-µm-thick free floating sections in PBS containing 1% Triton X-100 (Thermo Fisher Scientific, Waltham, MA, USA). Afterwards, sections were incubated with 0.1 m citrate buffer, pH 6, at 60°C for 5 min.

For detection of Tau phosphorylated epitopes, sections for CP13 and AT100 staining were treated with 70% formic acid for 7 min for antigen retrieval. Endogenous peroxidase (for detection of insulin receptors and AT100 staining) was inactivated by incubation of sections with 3% hydrogen peroxide in methanol for 2 h. Sections were then blocked with 5% BSA (Sigma, St Louis, MO, USA) and 5% normal goat serum (Invitrogen, Carlsbad, CA, USA) in 1% Triton X-100 for 3 h at room temperature. Primary antibodies were diluted in blocking solution and incubated with sections at 4°C for 16 h, followed by incubation with biotinylated secondary antibody for 2 h at room temperature, and then processed using the Vectastain Elite ABC reagent (Burlingame, CA, USA) according to the manufacturer’s instructions. The sections were washed in PBS and developed using DAB (DakoCytomation, Glostrup, Denmark) in chromogen solution, and counterstained with Harris’ hematoxylin (Merck, São Paulo, Brazil).. Slides were mounted with Entellan (Merck, São Paulo, Brazil) and imaged on a Zeiss Axio Observer Z1 microscope. Omission of primary antibody was routinely used to check for the absence of non-specific labeling (data not shown).

For immunofluorescence analysis, tissue autofluorescence was quenched by incubation with 0.06% potassium permanganate (Merck) for 10 min at room temperature. Sections were blocked in 5% BSA and 5% normal goat serum in 1% Triton X-100 for 3 h at room temperature. Primary antibodies were diluted in blocking solution and sections were incubated at 4°C for 16 h, followed by incubation with Alexa594- and Alexa488-conjugated secondary antibodies (1:2000) for 2 h at room temperature. Nuclei were stained with DAPI (Invitrogen, [Carlsbad](https://www.google.com.br/search?rlz=1c1rnkb_enbr485br485&espv=210&es_sm=122&q=carlsbad+california&stick=h4siaaaaaaaaagoovnz8bqmdgwshnxcxfq6-gulvrup8rhihif1kup6npzwdbkwfx5semjdzlvismz-hwrhkse1mksxnlcpjlsrwvfixcmjyfkmkn9zh7fzvj79boc8jap2rsvthaaaa&sa=x&ei=mpsmutx1bnk5kqfuw4g4cg&ved=0clgbejstkaiweq), [CA](https://www.google.com.br/search?rlz=1c1rnkb_enbr485br485&espv=210&es_sm=122&q=california&stick=h4siaaaaaaaaagoovnz8bqmdgwshnxcxfq6-gulvrup8rhihig2yz16opzwdbkwfx5semjdzlvismz-hwrhkse1mksxnlcpjlso-9guraisl_n-pxgdl-hla7v5v3dwjafqmskhhaaaa&sa=x&ei=mpsmutx1bnk5kqfuw4g4cg&ved=0clkbejstkamweq), USA) for 5 min. Immunostaining was carried out in parallel in slides from all animals. Slides were mounted with Prolong Gold Antifade with DAPI (Invitrogen) and imaged on a Zeiss Axio Observer Z1 microscope equipped with an Apotome module to minimize out-of-focus light. For synaptic puncta analyses, cells were imaged on a Leica confocal microscope. For CP13 immunohistochemistry, z-stack projections were acquired from regions of interest (total area: 7 µm for each image: *x* =0.06 µm/pixel, *y* = 0.06 µm/pixel, *z* = 0.28 µm/pixel).

**Electron microscopy**

The samples were washed three times with 0.1 m phosphate buffer (pH 7.4) and dehydrated with a graded acetone series. Uranyl acetate (1%) (Sigma, St. Louis, MO, USA) was added to the 70% ethanol (35 min immersion) to improve contrast. The sections were then embedded in POLYBED 812 resin (Electron Microscopy Science, PA, USA) and polymerized for 48 h at 60°C. Ultrathin sections were cut with an RMC ultramicrotome TX (RMC Boeckeler, CA, USA), collected on copper grids, stained with uranyl acetate and lead citrate, and imaged on a JEOL (JEM101) Transmission Electron Microscope (JEOL, Peabody, MA, USA). In brief, 50 images per animal were acquired randomly using a JEOL transmission EM (JEOL 1011) with a digitalizing image system (Gatan Inc, CA, USA). Synapses were quantified in the neuropil (i.e. avoiding the neuronal and glial somata and blood vessels). All synapses were counted in each electron micrograph, which represented ~7.4 µm^2^ of tissue (total ~370 µm^2^ per animal). Units presenting synaptic vesicles in the presynaptic element and pre- and post-synaptic membrane specializations visible with or without synaptic cleft evident were counted. Synaptic profiles touching the edges of electron micrographs were not counted.

**Western blotting**

Samples from the superior frontal gyrus of macaques were homogenized in RIPA buffer containing protease and phosphatase inhibitor cocktails and heated at 105°C for 20 min. Extracts were incubated on ice for 5 min, centrifuged at 14 000 × g for 30 min, and the supernatant was collected Protein concentration was determined using the enhanced BCA protein assay kit (Thermo Fisher Scientific). Samples (40 µg total protein/lane) were resolved on a 4–20% polyacrylamide gel with Tris/glycine/SDS (Sigma; Merck) buffer run at 125 V for 80 min at room temperature. For experiments with anti-Tau antibodies, the gel was electroblotted onto Odyssey nitrocellulose membrane using 25 mm Tris, 192 mm glycine, 20% (v/v) methanol (Merck), 0.02% SDS, pH 8.3, at 350 mA for 2 h at 4°C. Membranes were blocked with Odyssey blocking solution in Tris-buffered saline containing Tween 20 (TBS-T; 0.1% Tween 20 in 20 mm Tris–HCl, pH 7.5, 0.8% NaCl) for 1 h at room temperature. Primary antibodies (taupSer396, 1:100 and Tau-5, 1:100) were diluted in blocking solution/TBS and incubated with the membranes for 2 h at room temperature. After incubation with infrared labeled secondary antibodies goat anti-mouse IRDye 800 or goat anti-rabbit IRDye 800 IgG (H L; 1:10 000 in blocking buffer; Invitrogen) for 60 min, membranes were washed and scanned in an Odyssey Infrared System (Li-Cor Bioscience, Nebraska, USA).

**RNA extraction and quantitative real-time PCR analysis**

Hippocampi from vehicle-, AβO-injected, and/or liraglutide-treated mice were homogenized in 1 ml of Trizol (Thermo Fisher Scientific) and RNA extraction (SV Total RNA Isolation System, Promega) was performed according to the manufacturer’s instructions. Purity and amount of RNA were determined by the 260/280 nm absorbance ratio. Only preparations with ratios between 2.2 and 1.8 and no signs of RNA degradation were used. One microgram of RNA was used for cDNA synthesis using the SuperStrand III Reverse Transcriptase Kit (Thermo Fisher Scientific). Expression of genes of interest was analyzed by qPCR on an Applied Biosystems 7500 RT-PCR system using the Power SYBR kit (Applied Biosystems, Waltham, MA, USA). Actin was used as an endogenous control. Cycle threshold (Ct) values were used to calculate fold changes in gene expression using the 2^−ΔΔCt^ method. In all cases, reactions were performed in 15 µl reaction volumes. The oligonucleotide sequences for genes studied in this paper were: insulin receptor-α forward (5'ATGGGCTTCGGGAGAGGAT3'), insulin receptor-α reverse (5'GGATGTCCATACCAGGGCAC3'); *Actb* forward 5'GTCTTCCCCCTCCATCGTG3'), *Actb* reverse 5'AGGATGCCTCTCTTGCTCTG3').
