# Supplementary material for: The diabetes drug liraglutide reverses cognitive impairment in mice and attenuates insulin receptor and synaptic pathology in a non‐human primate model of Alzheimer's disease
Source: J Pathol. 2018 Apr 2;245(1):85–100. doi: 10.1002/path.5056 (PMC5947670; doi:10.1002/path.5056)
Supplement: Supplementary file 2 — Supplementary figure legends [file PATH-245-85-s002.doc]

**Supplementary figure legends**

**Figure S1. PKA activation is required for GLP-1 receptor-mediated prevention of AβO-induced synapse loss. (A)** Representative images of cultured hippocampal neurons exposed to 500 nm AβOs (or vehicle) for 3 h and immunolabeled for synaptophysin (green)/PSD-95 (red). Where indicated, neurons were pre-incubated with liraglutide (300 nm) or H-89 (10 µm) for 40 min. Scale bar = 60 µm. Insets show higher-magnification images of selected dendrite segments. Scale bar = 10 µm. Integrated immunoreactivities for synaptophysin **(B)**, PSD-95 **(C)** or co-localized synaptophysin/PSD-95 puncta **(D)**. Data are expressed as means ± SEM from three experiments from independent neuronal cultures (30 images analyzed per experimental condition per experiment). **p* < 0.05, one-way ANOVA followed by Bonferroni *post hoc* test. *P* value: In **B**: vehicle versus AβOs (*p =* 0.0073); AβOs versus Lira + AβOs (*p =* 0.0012); Lira + AβOs versus H89 + Lira + AβOs (*p =* 0.023); H89 versus Lira + AβOs (0.01); AβOs versus H89 (*p =* 0.04). In **C**: vehicle versus AβOs (*p =* 0.0006); AβOs versus Lira + AβOs (*p =* 0.0074); Lira + AβOs versus H89 + Lira + AβOs (*p =* 0.0014); H89 versus Lira + AβOs (0.0239); AβOs versus H89 (*p =* 0.051). In **D**: vehicle versus AβOs (*p =* 0.0009); AβOs versus Lira + AβOs (*p =* 0.012); Lira + AβOs versus H89 + Lira + AβOs (*p =* 0.03); H89 versus Lira + AβOs (0.0006); AβOs versus H89 (*p =* 0.045).

**Figure S2. Intracerebroventricular AβO injection or liraglutide treatment does not interfere with hippocampal insulin levels in mice.** Hippocampal insulin levels were measured 9 days after i.c.v. injection of AβOs (10 pmol) or vehicle. Prior to AβO injection, animals were pretreated for 7 days with saline or liraglutide (25 nmol/kg; i.p.; *n* = 5–6 animals per group). One-way ANOVA followed by Bonferroni *post hoc* test. *P* value: vehicle + saline versus AβO + saline (*p =* 0.8036); AβOs + saline versus vehicle + Lira (*p =* 0.8834); AβOs + saline versus Lira + AβOs (*p* > 0.9999).

**Figure S3. Effects of liraglutide on AβO-induced loss of insulin receptor in the frontal cortex, hippocampus, and amygdala of NHPs.** Representative images of frontal cortex, hippocampus, and amygdala of sham-operated, AβO-injected or liraglutide-treated AβO-injected NHPs (as indicated) immunolabeled for IRα (**A, C, E**) or IRβ (**B, D, F**). Scale bar = 50 µm.

**Figure S4. Liragutide attenuates AβO-induced synapse damage in the hippocampus and amygdala of NHPs.** Representative images from the dentate gyrus of sham-operated, AβO-injected or liraglutide-treated AβO-injected NHPs (as indicated) immunolabeled for synaptophysin (**A**) or PSD-95 (**D**). Merged images are shown in (**G**). Nuclear staining (DAPI) is shown in blue. Scale bar = 30 µm. Graphs represent the number of punctae per unit area (relative to sham-operated NHPs) for synaptophysin (**B, C**) or PSD-95 (**E, F**) in different brains regions (as indicated). For representative images from amygdala see **Figure S5. (H, I)** Number of co-localized synaptophysin/PSD-95 immunoreactive punctae per unit area (relative to sham-operated NHPs). Data are expressed as means ± SEM. (*n* = 3 sham-operated, *n* = 4 AβO-injected NHPs, *n* = 2 liraglutide-treated AβO-injected NHPs). Data are expressed as means ± SEM. One-way ANOVA followed by Bonferroni *post hoc* test. *P* value: In **B**: sham versus AβOs: *p =* 0.0009; AβOs versus Lira + AβOs: *p =* 0.09. In **C**: sham versus AβOs: *p =* 0.001; AβOs versus Lira + AβOs: *p =* 0.0049. In **E**: sham versus AβOs: *p =* 0.0003; AβOs versus Lira + AβOs: *p =* 0.03. In **F**: sham versus AβOs: *p =* 0.0003; AβOs versus Lira + AβOs: *p =* 0.0251. In **H**: sham versus AβOs: *p =* 0.0001; AβOs versus Lira + AβOs: *p =* 0.0042. In **I**: sham versus AβOs: *p =* 0.0001; AβOs versus Lira + AβOs: *p =* 0.002.

**Figure S5. Representative images of synapse densities in the frontal cortex and amygdala of NHPs.** Representative images from the frontal cortex or amygdala of sham-operated, AβO-injected or liraglutide-treated AβO-injected NHPs immunolabeled for synaptophysin (green) **(A, C)** and PSD-95 **(B, D)** (red). Merged images from amygdala areas shown in **E**. Scale bar = 30 µm

**Figure S6. Representative images of hippocampus and amygdala immunolabeled for AT100 or CP13 in the NHPs.** Representative images of hippocampus and amygdala of sham-operated, AβO-injected or liraglutide-treated AβO-injected NHPs (as indicated) immunolabeled for AT100 **(A-B)** or CP13 **(C-D)**. Scale bar = 50 µm in (**A-B)** and 20 µm in (**C-D)**. For CP13, z-stack projections were performed. Nuclear staining (DAPI) is shown in blue.
