# Supplementary figures and images for: The diabetes drug liraglutide reverses cognitive impairment in mice and attenuates insulin receptor and synaptic pathology in a non‐human primate model of Alzheimer's disease
Source: J Pathol. 2018 Apr 2;245(1):85–100. doi: 10.1002/path.5056 (PMC5947670; doi:10.1002/path.5056)

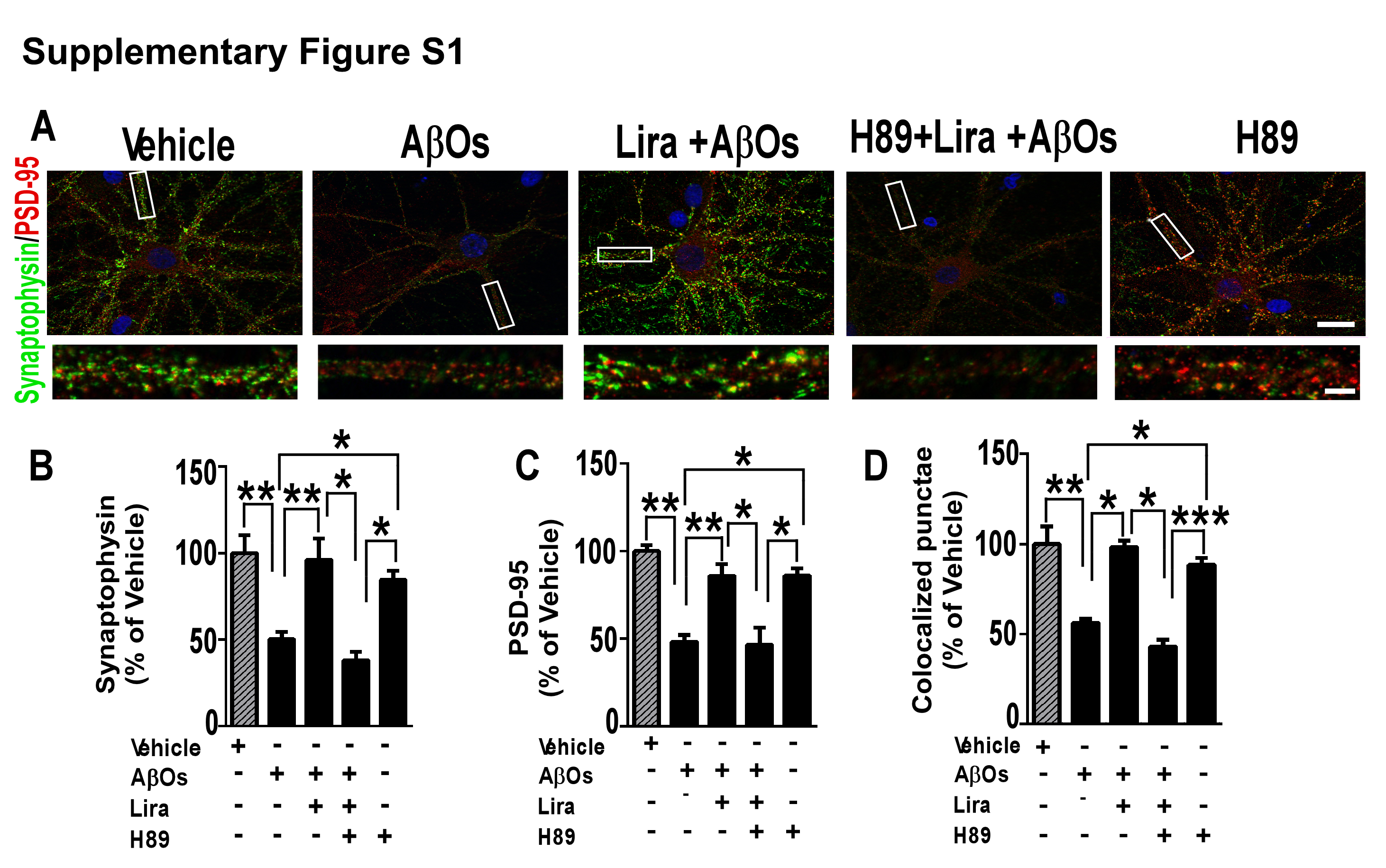

Supplement: Supplementary file 3 — Figure S1. PKA activation is required for GLP‐1 receptor‐mediated prevention of AβO‐induced synapse loss. (A) Representative images of cultured hippocampal neurons exposed to 500 nm AβOs (or vehicle) for 3 h and immunolabeled for synaptophysin (green)/PSD‐95 (red). Where indicated, neurons were pre‐incubated with liraglutide (300 nm) or H‐89 (10 μm) for 40 min. Scale bar = 60 μm. Insets show higher‐magnification images of selected dendrite segments. Scale bar = 10 μm. Integrated immunoreactivities for synaptophysin (B), PSD‐95 (C) or co‐localized synaptophysin/PSD‐95 puncta (D). Data are expressed as means ± SEM from three experiments from independent neuronal cultures (30 images analyzed per experimental condition per experiment). *p < 0.05, one‐way ANOVA followed by Bonferroni post hoc test. P value: In B: vehicle versus AβOs (p = 0.0073); AβOs versus Lira + AβOs (p = 0.0012); Lira + AβOs versus H89 + Lira + AβOs (p = 0.023); H89 versus Lira + AβOs (0.01); AβOs versus H89 (p = 0.04). In C: vehicle versus AβOs (p = 0.0006); AβOs versus Lira + AβOs (p = 0.0074); Lira + AβOs versus H89 + Lira + AβOs (p = 0.0014); H89 versus Lira + AβOs (0.0239); AβOs versus H89 (p = 0.051). In D: vehicle versus AβOs (p = 0.0009); AβOs versus Lira + AβOs (p = 0.012); Lira + AβOs versus H89 + Lira + AβOs (p = 0.03); H89 versus Lira + AβOs (0.0006); AβOs versus H89 (p = 0.045). [file PATH-245-85-s003.tif]

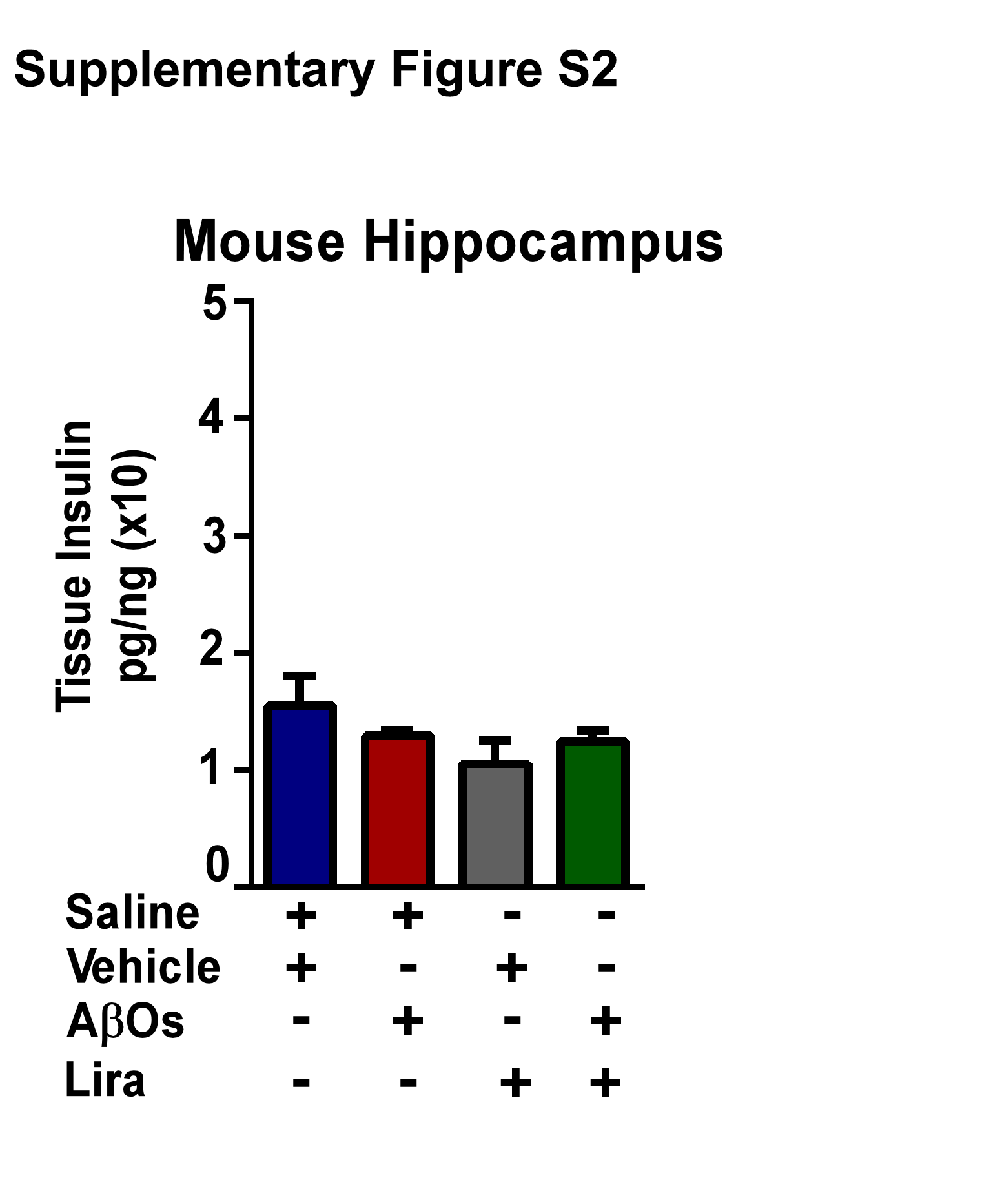

Supplement: Supplementary file 4 — Figure S2. Intracerebroventricular AβO injection or liraglutide treatment does not interfere with hippocampal insulin levels in mice. Hippocampal insulin levels were measured 9 days after i.c.v. injection of AβOs (10 pmol) or vehicle. Prior to AβO injection, animals were pretreated for 7 days with saline or liraglutide (25 nmol/kg; i.p.; n = 5–6 animals per group). One‐way ANOVA followed by Bonferroni post hoc test. P value: vehicle + saline versus AβO + saline (p = 0.8036); AβOs + saline versus vehicle + Lira (p = 0.8834); AβOs + saline versus Lira + AβOs (p > 0.9999). [file PATH-245-85-s004.tif]

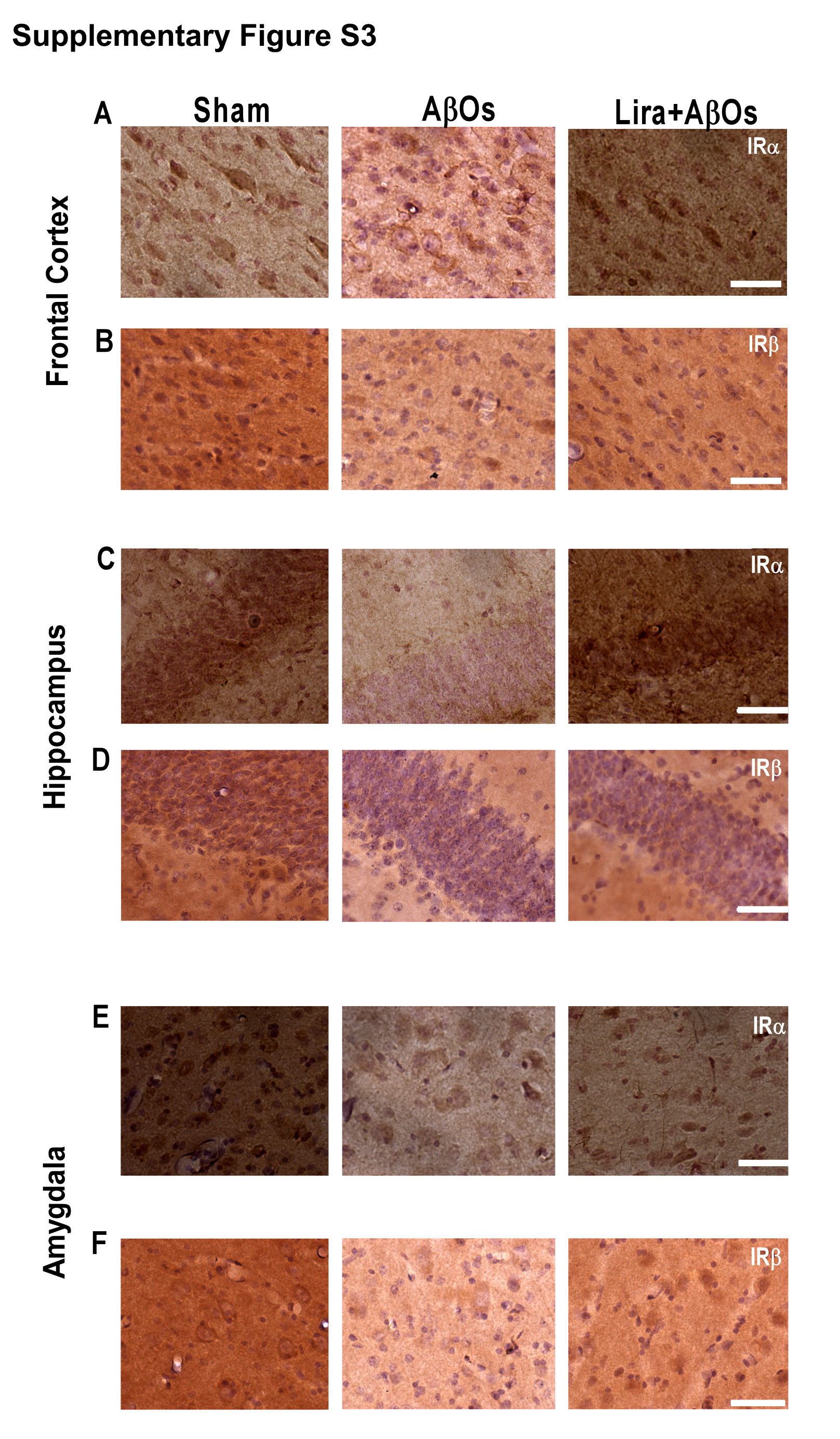

Supplement: Supplementary file 5 — Figure S3. Effects of liraglutide on AβO‐induced loss of insulin receptor in the frontal cortex, hippocampus, and amygdala of NHPs. Representative images of frontal cortex, hippocampus, and amygdala of sham‐operated, AβO‐injected or liraglutide‐treated AβO‐injected NHPs (as indicated) immunolabeled for IRα (A, E, I) or IRβ (C, G, K). Scale bar = 50 μm. Quantification of integrated optical density for immunoreactivities of IRα or IRβ in the frontal cortex (B and D, respectively), hippocampus (F and H, respectively), and amygdala (J and L, respectively). Data are expressed as means ± SEM; n = 3 sham‐operated, n = 4 AβO‐injected, n = 2 liraglutide‐treated AβO‐injected NHPs. One‐way ANOVA followed by Bonferroni post hoc test. P value: In B: sham versus AβOs: p = 0.001; AβOs versus Lira + AβOs: p = 0.02. In D: sham versus AβOs: p = 0.01; AβOs versus Lira + AβOs: p = 0.14. In F: sham versus AβOs: p = 0.02; AβOs versus Lira + AβOs: p = 0.007. In H: sham versus AβOs: p = 0.002; AβOs versus Lira + AβOs: p = 0.97. In J: sham versus AβOs: p = 0.0432; AβOs versus Lira + AβOs: p = 0.99. In L: sham versus AβOs: p = 0.004; AβOs versus Lira + AβOs: p = 0.44. [file PATH-245-85-s005.tif]

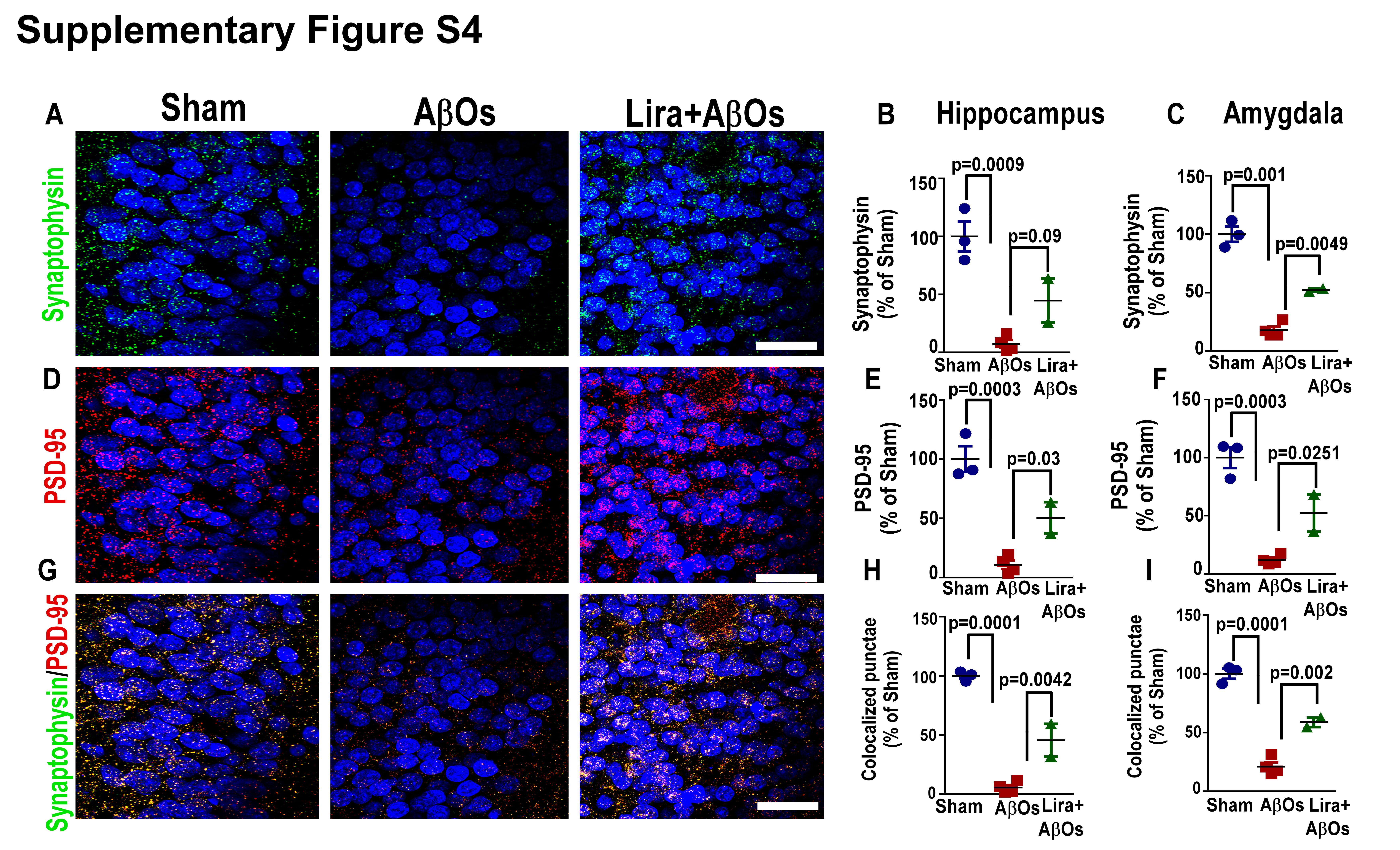

Supplement: Supplementary file 6 — Figure S4. Liragutide attenuates AβO‐induced synapse damage in the hippocampus and amygdala of NHPs. Representative images from the dentate gyrus of sham‐operated, AβO‐injected or liraglutide‐treated AβO‐injected NHPs (as indicated) immunolabeled for synaptophysin (A) or PSD‐95 (D). Merged images are shown in (G). Nuclear staining (DAPI) is shown in blue. Scale bar = 30 μm. Graphs represent the number of punctae per unit area (relative to sham‐operated NHPs) for synaptophysin (B, C) or PSD‐95 (E, F) in different brains regions (as indicated). For representative images from amygdala see Figure S5. (H, I) Number of co‐localized synaptophysin/PSD‐95 immunoreactive punctae per unit area (relative to sham‐operated NHPs). Data are expressed as means ± SEM. (n = 3 sham‐operated, n = 4 AβO‐injected NHPs, n = 2 liraglutide‐treated AβO‐injected NHPs). Data are expressed as means ± SEM. One‐way ANOVA followed by Bonferroni post hoc test. P value: In B: sham versus AβOs: p = 0.0009; AβOs versus Lira + AβOs: p = 0.09. In C: sham versus AβOs: p = 0.001; AβOs versus Lira + AβOs: p = 0.0049. In E: sham versus AβOs: p = 0.0003; AβOs versus Lira + AβOs: p = 0.03. In F: sham versus AβOs: p = 0.0003; AβOs versus Lira + AβOs: p = 0.0251. In H: sham versus AβOs: p = 0.0001; AβOs versus Lira + AβOs: p = 0.0042. In I: sham versus AβOs: p = 0.0001; AβOs versus Lira + AβOs: p = 0.002. [file PATH-245-85-s006.tif]

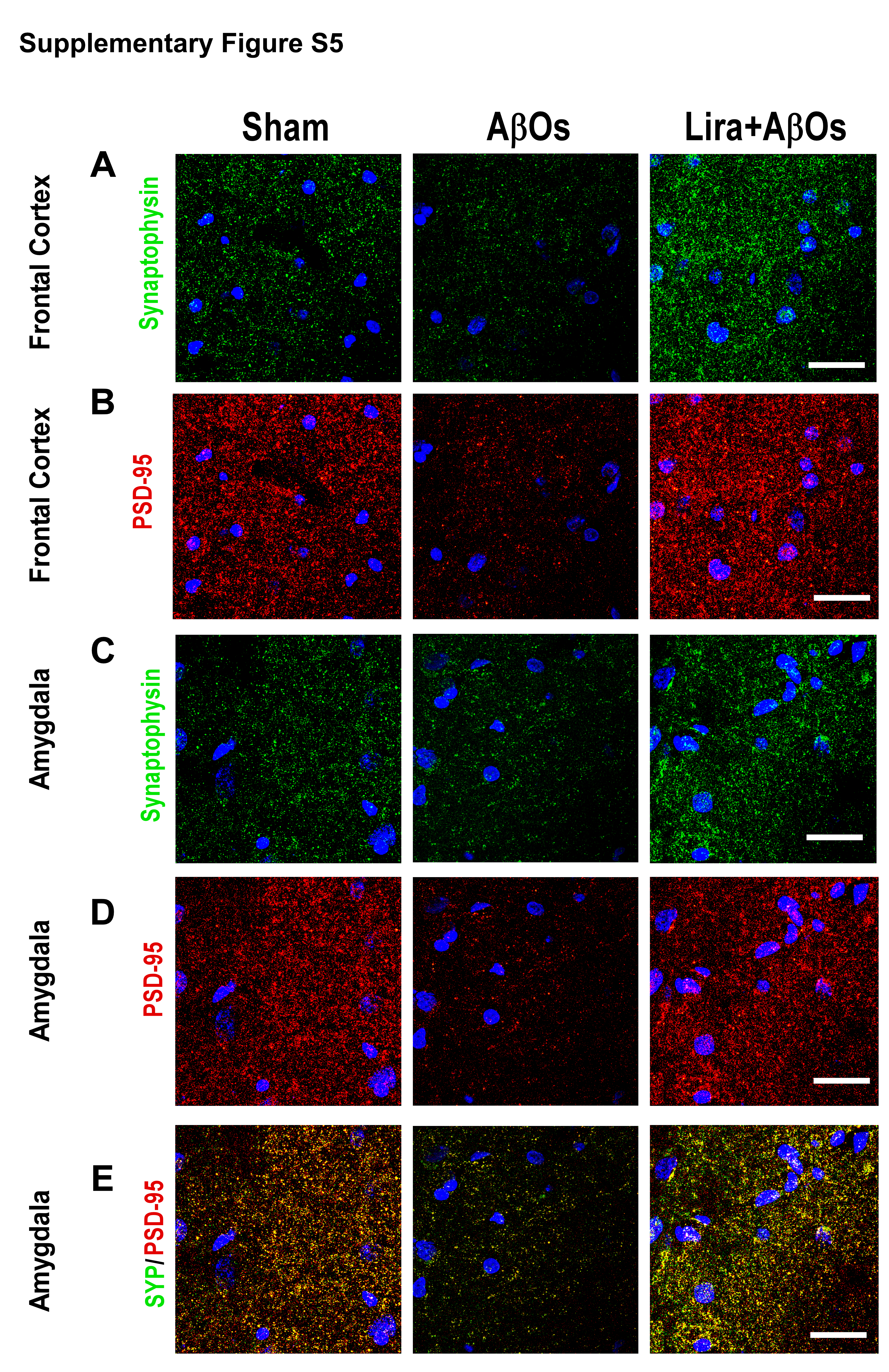

Supplement: Supplementary file 7 — Figure S5. Representative images of synapse densities in the frontal cortex and amygdala of NHPs. Representative images from the frontal cortex or amygdala of sham‐operated, AβO‐injected or liraglutide‐treated AβO‐injected NHPs immunolabeled for synaptophysin (green) (A, C) and PSD‐95 (B, D) (red). Merged images from amygdala areas shown in E. Scale bar = 30 μm [file PATH-245-85-s007.tif]

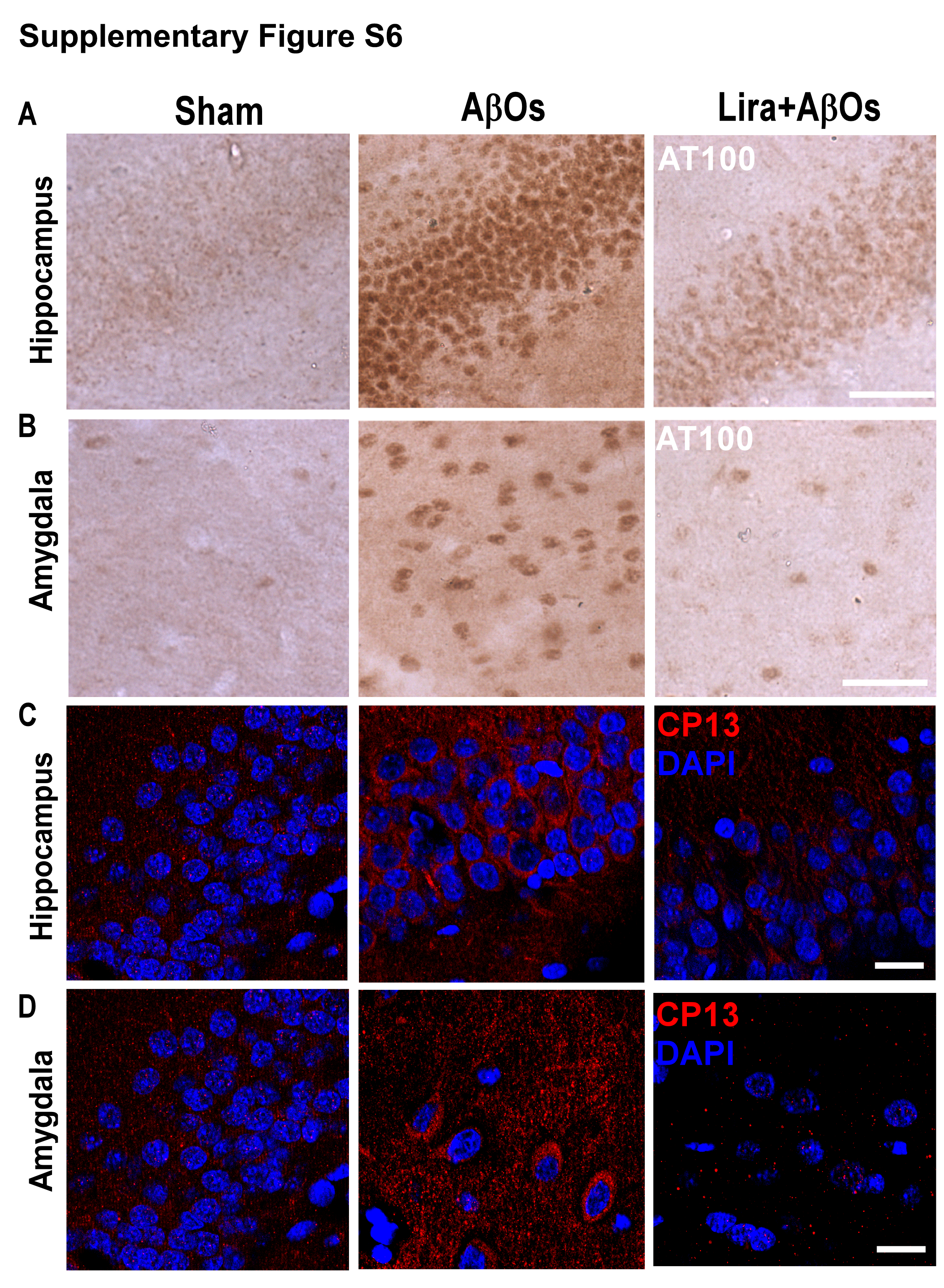

Supplement: Supplementary file 8 — Figure S6. Representative images of hippocampus and amygdala immunolabeled for AT100 or CP13 in the NHPs. Representative images of dentate gyrus and amygdala of sham‐operated, AβO‐injected or liraglutide‐treated AβO‐injected NHPs (as indicated) immunolabeled for AT100 (A) or CP13 (B). Scale bar = 50 μm in A and 20 μm in B. For CP13, z‐stack projections were performed. Nuclear staining (DAPI) is shown in blue. [file PATH-245-85-s008.tif]
