# Supplementary material for: The diabetes drug liraglutide reverses cognitive impairment in mice and attenuates insulin receptor and synaptic pathology in a non‐human primate model of Alzheimer's disease
Source: J Pathol. 2018 Apr 2;245(1):85–100. doi: 10.1002/path.5056 (PMC5947670; doi:10.1002/path.5056)
Supplement: Supplementary file 9 — Table S1. List of the primary antibodies used [file PATH-245-85-s009.doc]

**Table S1.** List of the primary antibodies used

| **Antibody** | **Supplier** | **Code** | **Dilution** | **Host** |
| --- | --- | --- | --- | --- |
| GluA1 | Santa Cruz Biotechnology, Santa Cruz, CA, USA | Sc-13152 | 1:200 | Mouse monoclonal |
| GluA2 | Santa Cruz | Sc-7611 | 1:100 | Goat polyclonal |
| GluN1 | Santa Cruz | Sc-9058 | 1:200 | Rabbit polyclonal |
| GluN2B | BD Transduction Laboratories, San Jose, CA, USA | 610417 | 1:200 | Rabbit polyclonal |
| PSD-95 | Santa Cruz | Sc-28941 | 1:200 | Rabbit polyclonal |
| Synaptophysin | Abcam, Cambridge, UK | ab14692 | 1:1,000 | Mouse monoclonal |
| IR-α | Santa Cruz | Sc-710 | 1:200 | Rabbit polyclonal |
| IR-β | Santa Cruz | Sc-711 | 1:200 | Rabbit polyclonal |
| Tau-pSer^396^  (recognizes phosphorylation of Tau at serine residue 396) | Santa Cruz | Sc-101815 | 1:200 | Rabbit polyclonal |
| AT-100  (recognizes phosphorylation of Tau at serine residue 212 and threonine residue 214) | Thermo Scientific Pierce Protein Research Products, Waltham, MA, USA | MN1060 | 1:70 | Rabbit polyclonal |
| CP13  (recognizes phosphorylation of Tau at serine residue 202) | * | * | 1:200 | Rabbit polyclonal |
| NU4 | Produced by Dr William Klein’s laboratory, Northwestern University, USA | – | 1:2000 | Mouse monoclonal |

*CP13 was a generous gift from Dr Peter Davies (Albert Einstein College of Medicine, Bronx, NY, USA).
