# Supplementary material for: The diabetes drug liraglutide reverses cognitive impairment in mice and attenuates insulin receptor and synaptic pathology in a non‐human primate model of Alzheimer's disease
Source: J Pathol. 2018 Apr 2;245(1):85–100. doi: 10.1002/path.5056 (PMC5947670; doi:10.1002/path.5056)
Supplement: Supplementary file 10 — Table S2. List of the secondary antibodies used [file PATH-245-85-s010.doc]

**Table S2.** List of the secondary antibodies used

| **Antibody** | **Supplier** | **Code** | **Dilution** |
| --- | --- | --- | --- |
| Goat anti-mouse IgG (H+L) secondary antibody, Alexa Fluor® 488 conjugate | Thermo Fisher Scientific, Waltham, MA, USA | A-11001 | 1:2000 |
| Goat anti-mouse IgG (H+L) secondary antibody, Alexa Fluor® 555 conjugate  Donkey anti-rabbit IgG secondary antibody, Alexa Fluor® 594 conjugate | Thermo Fisher Scientific  Thermo Fisher Scientific | A-21422  R37119 | 1:2000  1:2000 |
| Donkey anti-goat IgG (H+L) secondary antibody, Alexa Fluor® 555 conjugate | Thermo Fisher Scientific | A-21432 | 1:2000 |
| Biotinylated goat anti-mouse  IgG antibody | Vector Laboratories Inc, Burlingame, CA, USA | BA-9200 | 1:200 |
| Biotinylated goat anti-rabbit  IgG antibody | Vector Laboratories Inc | BA-1000 | 1:200 |
