# Supplementary material for: The diabetes drug liraglutide reverses cognitive impairment in mice and attenuates insulin receptor and synaptic pathology in a non‐human primate model of Alzheimer's disease
Source: J Pathol. 2018 Apr 2;245(1):85–100. doi: 10.1002/path.5056 (PMC5947670; doi:10.1002/path.5056)
Supplement: Supplementary file 11 — Table S3. Nomenclature for specific brain regions analyzed following intracerebroventricular injections of AβOs in NHPs [file PATH-245-85-s011.doc]

**Table S3.** Nomenclature for specific brain regions analyzed following intracerebroventricular injections of AβOs in NHPs

| **Nomenclature** | **Region analyzed** |
| --- | --- |
| Frontal cortex | Superior frontal gyrus layers 3, 4, and 5 |
| Hippocampus | Dentate gyrus |
| Amygdalar complex | Amygdaloid nuclear complex: accessory basal amygdaloid nucleus, basal amygdaloid nucleus, basolateral nuclear group of amygdala |
